# Multilocus Sequence Typing as a Replacement for Serotyping in *Salmonella enterica*

**DOI:** 10.1371/journal.ppat.1002776

**Published:** 2012-06-21

**Authors:** Mark Achtman, John Wain, François-Xavier Weill, Satheesh Nair, Zhemin Zhou, Vartul Sangal, Mary G. Krauland, James L. Hale, Heather Harbottle, Alexandra Uesbeck, Gordon Dougan, Lee H. Harrison, Sylvain Brisse, the S. enterica MLST study group

**Affiliations:** 1 Environmental Research Institute and Department of Microbiology, University College Cork, Cork, Ireland; 2 Max-Planck Institute for Infection Biology, Berlin, Germany; 3 The Wellcome Trust Sanger Institute, Wellcome Trust Genome Campus, Cambridge, United Kingdom; 4 Health Protection Agency, Centre for Infection, London, United Kingdom; 5 Institut Pasteur, Paris, France; 6 Infectious Diseases Epidemiology Research Unit, University of Pittsburgh School of Medicine and Graduate School of Public Health, Pittsburgh, Pennsylvania, United States of America; 7 Center for Veterinary Medicine, U. S. Food and Drug Administration, Derwood, Maryland, United States of America; 8 Institute of Medical Microbiology, Immunology, and Hygiene, University of Cologne, Cologne, Germany; New York Medical College, United States of America

## Abstract

*Salmonella enterica* subspecies *enterica* is traditionally subdivided into serovars by serological and nutritional characteristics. We used Multilocus Sequence Typing (MLST) to assign 4,257 isolates from 554 serovars to 1092 sequence types (STs). The majority of the isolates and many STs were grouped into 138 genetically closely related clusters called eBurstGroups (eBGs). Many eBGs correspond to a serovar, for example most Typhimurium are in eBG1 and most Enteritidis are in eBG4, but many eBGs contained more than one serovar. Furthermore, most serovars were polyphyletic and are distributed across multiple unrelated eBGs. Thus, serovar designations confounded genetically unrelated isolates and failed to recognize natural evolutionary groupings. An inability of serotyping to correctly group isolates was most apparent for Paratyphi B and its variant Java. Most Paratyphi B were included within a sub-cluster of STs belonging to eBG5, which also encompasses a separate sub-cluster of Java STs. However, diphasic Java variants were also found in two other eBGs and monophasic Java variants were in four other eBGs or STs, one of which is in subspecies *salamae* and a second of which includes isolates assigned to Enteritidis, Dublin and monophasic Paratyphi B. Similarly, Choleraesuis was found in eBG6 and is closely related to Paratyphi C, which is in eBG20. However, Choleraesuis var. Decatur consists of isolates from seven other, unrelated eBGs or STs. The serological assignment of these Decatur isolates to Choleraesuis likely reflects lateral gene transfer of flagellar genes between unrelated bacteria plus purifying selection. By confounding multiple evolutionary groups, serotyping can be misleading about the disease potential of *S. enterica*. Unlike serotyping, MLST recognizes evolutionary groupings and we recommend that *Salmonella* classification by serotyping should be replaced by MLST or its equivalents.

## Author Summary

Microbiologists have used serological and nutritional characteristics to subdivide pathogenic bacteria for nearly 100 years. These subdivisions in *Salmonella enterica* are called serovars, some of which are thought to be associated with particular diseases and epidemiology. We used MultiLocus Sequence-based Typing (MLST) to identify clusters of *S. enterica* isolates that are related by evolutionary descent. Some clusters correspond to serovars on a one to one basis. But many clusters include multiple serovars, which is of public health significance, and most serovars span multiple, unrelated clusters. Despite its broad usage, serological typing of *S. enterica* has resulted in confusing systematics, with a few exceptions. We recommend that serotyping for strain discrimination of *S. enterica* be replaced by a DNA-based method, such as MLST. Serotyping and other non-sequence based typing methods are routinely used for detecting outbreaks and to support public health responses. Moving away from these methods will require a major shift in thinking by public health microbiology laboratories as well as national and international agencies. However, a transition to the routine use of MLST, supplemented where appropriate by even more discriminatory sequence-based typing methods based on entire genomes, will provide a clearer picture of long-term transmission routes of *Salmonella*, facilitate data transfer and support global control measures.

## Introduction

**Figure 1:**
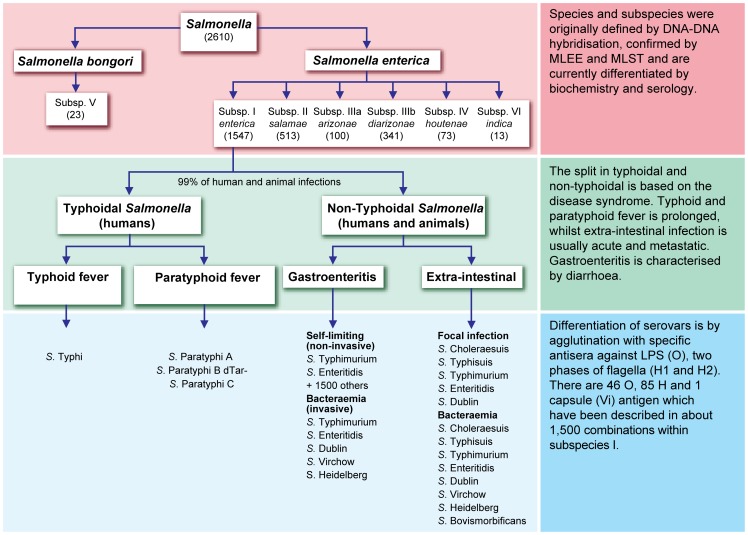
General overview of the current classification of *Salmonella enterica*.

For over 70 years, epidemiological investigations of *Salmonella* that infect humans and animals have depended on serotyping, the binning of isolates into serovars [1], [2]. *Salmonella* serotyping depends on specific agglutination reactions with adsorbed antisera that are specific for epitopes (‘factors’) within either lipopolysaccharide (O antigen; encoded by *rfb* genes) or one of the two, alternate flagellar antigens (phases 1 and 2 of H antigen, encoded by *fliC* and *fljB*). Various combinations of 46 O antigens and 85 H antigens have resulted in ∼1,500 serovars within *S. enterica* subspecies *enterica* and ∼1000 in the other subspecies of *S. enterica* plus *S. bongori* (Fig. 1) [2].

The use of serotyping within *Salmonella* as a typing method is so widely accepted that governmental agencies have formulated guidelines intended to reduce human salmonellosis by targeting Typhimurium, Enteritidis and three other common serovars in domesticated animals (European Union EC Regulation 2160/2003 of 12/12/2003). Such regulations implicitly assume that serovars are associated with a particular disease potential [3], [4], an assumption that is also suggested by some of their names, e.g. Abortusequi, Abortusovis and Choleraesuis. These designations reflect a medical microbiological tradition of assigning distinctive taxonomic designations to microorganisms that are associated with particular diseases or hosts. However, this tradition is not necessarily warranted from an evolutionary perspective, as illustrated by the following examples. For some taxa, species designations have been used to designate genetically monomorphic clones of a broader species with a different pathogenic potential, e.g. the clone of *Yersinia pseudotuberculosis* that is called *Y. pestis* [5], the host-specific ecotypes of the *Mycobacterium tuberculosis* complex that are designated *M. bovis*, *M. microti*, *M. pinnipedii* and *M. caprae* [6], or the isolates of *Escherichia coli* that have been assigned to multiple species of the genus *Shigella* [7]. In other cases, taxonomic designations have grouped members of paraphyletic groups of microorganisms because they cause similar diseases, such as the anthrax toxin-producing variants of *Bacillus cereus* that are designated *Bacillus anthracis* [8]. That all isolates of an individual serovar of *S. enterica* share a common phylogenetic ancestry should therefore be considered to represent a working hypothesis that requires confirmation. Similarly, a supposed host and/or disease specificity needs to be confirmed by genetically informative methods with isolates from diverse geographical regions. These working hypotheses has been confirmed for serovar Typhi, which corresponds to a genetically monomorphic, recently evolved clone that causes typhoid fever in humans [9]–[11]. In contrast, multiple, discrete lineages have been identified within serovar Newport [12]. Close genetic relatedness and a monolithically uniform association with host/disease specificity remain to be demonstrated for most other serovars, especially because only few of them have yet been investigated in detail.

Serovar designations are widely used for epidemiological purposes due to the belief that they are discriminatory, and because serovars represent a globally understandable form of communication. However, as noted by McQuiston *et al.* [13], [14], serotyping has multiple disadvantages, including low throughput, high expense, and a requirement for considerable expertise as well as numerous antibodies made by immunizing rabbits. As a result, various molecular methods have been proposed as potential alternatives to serotyping for subdividing *Salmonella* (and other microbes) [15], [16], ranging from PFGE (Pulsed-Field Gel Electrophoresis) [17], [18] through to MLVA (MultiLocus Variable number of tandem repeats Analysis) [19], [20]. These methods are possibly useful for recognizing a common source of microorganisms from a single outbreak [21], but they are inappropriate for reliable assignments of isolates to one of the 2,500 *S. enterica* serovars. Still other attempts have been made to develop DNA-sequence based equivalents of serotyping [22]–[26], including the detection of particular single nucleotide polymorphisms (SNPs) within flagellar antigens [13], [14]. This approach shares with serotyping the assumption that serotyping reflects genetic relatedness or disease specificity, which needs not be generally true [12]. For example, genes encoding antigenic epitopes can be imported by horizontal genetic exchange and homologous recombination from unrelated lineages. As a result, genetically related serovars such as Heidelberg and Typhimurium possess very different *fliC* alleles whereas genetically distinct serovars can possess nearly identical alleles [27]. Thus, replacing serological determination by serotype-based molecular assays would maintain a system that does not necessarily reflect genetic relatedness. Furthermore, some serovar designations will need revision because they distinguish between minor antigenic variants of organisms that are genetically very similar, e.g. Dublin and Rostock [28] or Paratyphi A and Sendai [29].

We recommend another approach, namely using neutral markers to identify genetically related clusters of *S. enterica*. Serovar designations that reflect such groupings could be preserved, and possibly be detected by informative SNPs in those neutral markers, whereas other serovars need to be revised or possibly eliminated. Twenty years ago, a valiant attempt was made to identify natural groupings within *S. enterica* on the basis of MultiLocus Enzyme Electrophoresis (MLEE) [29]–[31]. MLEE data identified multiple monophyletic lineages that corresponded to individual serovars. Problematically, most serovars that were examined included exceptional isolates that were unrelated to the main lineage, and some serovars were composed of multiple, genetically unrelated lineages rather than one predominant lineage. MLEE was never generally accepted by microbiologists and these observations have not influenced the general use of serovar designations.

Instead of MLEE, a sequence-based alternative, MultiLocus Sequence Typing (MLST), has gained broad acceptance for many microbial species [32]. MLST is based on similar principles to MLEE, but has greater discrimination and is more objective because it is based on sequences of multiple housekeeping gene fragments rather than electrophoretic migration of proteins. Of equal importance, MLST schemes are community efforts because the data are publicly available online (http://pubmlst.org/databases.shtml) and data can be entered from decentralized sources. Isolates that possess identical alleles for all gene fragments are assigned to a common Sequence Type (ST), and STs that share all but one or two alleles are grouped into ST-based clonal complexes [33] on the basis of eBurst [34]. An MLST scheme involving seven housekeeping gene fragments was developed for the analysis of serovar Typhi [9], and subsequently tested with 110 isolates from 25 serovars of *S. enterica* subspecies *enterica* [35], most of which were from Selander's SARB collection of reference strains for MLEE [30]. Subsequent analyses have used this scheme to survey serovars Newport [12], [36] and Typhimurium [37]–[39], as well as smaller numbers of isolates of various serovars from wild animals in Australia [40] and the mesenteric lymph nodes of cattle in Canada [41]. The same scheme has also been used to survey the genetic properties of antibiotic-resistant isolates among a global sample of various serovars [42]. These initial results suggested that MLST often correlates with serovar, with some exceptions. If this inference were correct, it would be advisable to replace serotyping by MLST for routine epidemiological purposes. We therefore embarked on a major, decentralized effort to test this hypothesis.

We investigated isolates from diverse hosts, both diseased and healthy, as well as from the environment. We screened isolates from all continents and deliberately included representatives of rare serovars as well as unusual monophasic and diphasic variants from reference collections. All this data was submitted to a publically accessible MLST database (http://mlst.ucc.ie/mlst/dbs/Senterica). In April, 2011, that database included 4,257 isolates (Table S1) from 554 serovars of *S. enterica* subspecies *enterica* that had been assigned to 1,092 STs. The database also contained 436 isolates from the other *S. enterica* subspecies as well as *Salmonella bongori*, whose properties will be described elsewhere, as will analyses of associations with host or geography.

Here we describe the population structure of subspecies *enterica* on the basis of MLST, examine the extent of congruence between serotyping and MLST clusters, and conclude that serotyping of *S. enterica* should be replaced by MLST.

## Results

Many *Salmonella* STs cluster together in discrete groups, which we refer to as eBGs (eBurstGroups). We chose the designation eBG rather than “Clonal Complex” or “ST Complex” because Clonal Complex implies clonality [43], whereas homologous recombination between unrelated lineages is frequent in *S. enterica* [12], [44], [45], and ST Complex does not specify a grouping algorithm. Following the recommendations by Feil *et al.* [46], [47], we designated as an eBG all groups of two or more STs that were connected by pair-wise identity at six of the seven gene fragments, i.e. they shared six of the seven alleles that defined the ST. As the MLST database has grown, multiple singleton STs containing multiple isolates have formed eBG clusters *via* the incremental identification of novel, related STs. We therefore also designated ungrouped singleton STs as eBGs when they contained 10 or more isolates. Finally, a few existing eBGs were expanded to include singleton STs that shared five identical alleles (double locus variants; DLVs) as well as a common serovar. Based on these criteria, 3,550 of the 4,257 isolates were assigned to a total of 138 eBGs, containing between 580 isolates in multiple STs and two isolates in two STs (Table S2).

### eBGs are natural clusters of genetically related isolates

**Figure 2:**
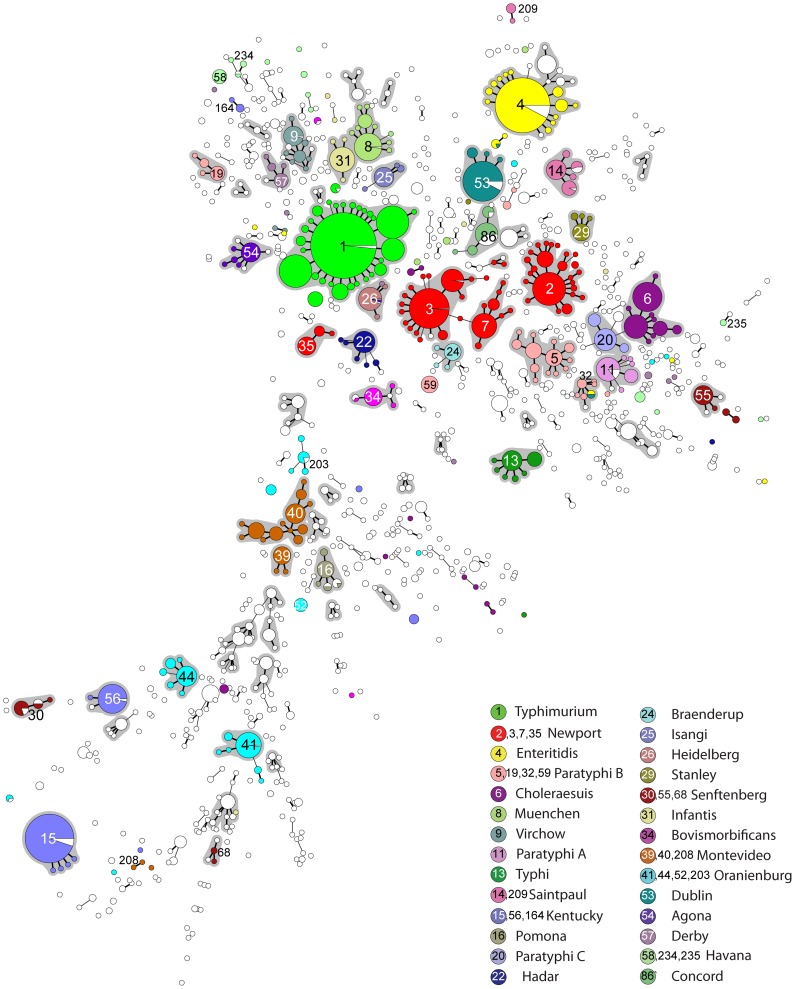
Minimal spanning tree (MSTree) of MLST data on 4257 isolates of *S. enterica* subspecies *enterica*. Each circle corresponds to one of 1,095 STs, whose size is proportional to the number of isolates. The topological arrangement within the MSTree is dictated by its graphic algorithm, which uses an iterative network approach to identify sequential links of increasing distance (fewer shared alleles), beginning with central STs that contain the largest numbers of isolates. As a result, singleton STs are scattered throughout the MSTree proximal to the first node that was encountered with shared alleles, even if equal levels of identity exist to other nodes that are distant within the MSTree. The figure only show links of six identical gene fragments (SLVs; thick black line) and five identical gene fragments (DLVs; thin black line) because these correlate with eBGs, which are indicated by grey shading. The serovar associated with most of the isolates in each eBG or singleton ST is indicated by color coding for the 28 most frequent serovars (see legend at lower right). Within each ST, isolates of a different serovar or for which information is lacking are shown in white, except for monophasic variants.

We initially recognized the existence of eBGs by visual examination of a minimal spanning tree (MSTree) of STs connected by the numbers of shared alleles. The MSTree of subspecies *enterica* shows multiple starburst-like clusters (Fig. 2), which in large part correspond to eBGs as defined here. Similar to eBurst groups in other species, most clusters radiate from a central node which contains numerous isolates, a phenomenon which is usually interpreted as representing monophyletic lineages of STs that have evolved from a single founder node [34]. We deferred interpretations on evolutionary history within eBGs, including the identification of founders, until genomic studies of historically representative isolates have been conducted, and therefore arbitrarily assigned an otherwise uninformative, unique number to each eBG.

Historically, MLEE data of *S. enterica* were interpreted on the basis of phylogenetic trees [29]–[31]. Trees attempt to depict genealogies (vertical descent from a common ancestor), and can be confounded by homologous recombination between unrelated lineages, a common occurrence in *S. enterica* [44], [45]. Indeed, only one higher level population structure with strong statistical support has been identified within subspecies *enterica*; this structure has been referred to as Clade B [40], [44], [48] or Lineage 3 [45]. We confirmed the existence of Lineage 3 in our large dataset by a BAPS [49] cluster analysis of the allelic differences between STs using an upper bound of 2–7 clusters (Fig. S2). Similar results were obtained with concatenated sequences for all seven gene fragments regardless of upper bound, or when using Structure [50].

**Figure 3:**
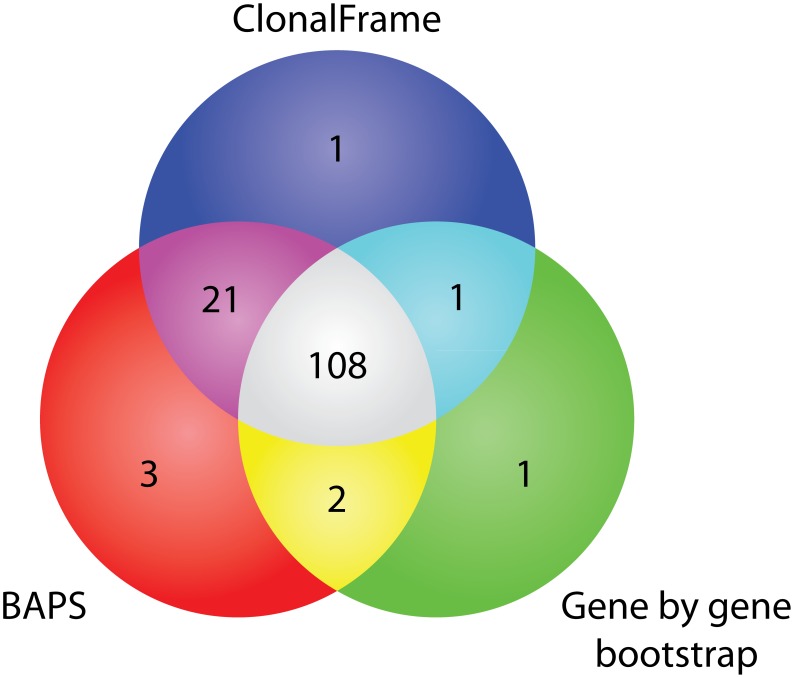
Venn diagram of numbers of eBGs whose STs were assigned to a single cluster by ClonalFrame, BAPS and gene by gene bootstrapping. Other details are in Table 1.

In order to assess the robustness of our eBG classification, we investigated the fine structure of subspecies *enterica* by three additional, independent clustering methods. Firstly, we analyzed concatenated sequences with ClonalFrame [51], which determines tree topologies after stripping signals of lateral gene transfer and homologous recombination. ClonalFrame identified 163 lineages containing more than one ST (Table 1), each of which coalesced far from the root (Fig. S3). This result provides further support for the conclusion [44], [45] that there is little deep phylogenetic signal within the MLST genes. Secondly, we analyzed the sequence data by a gene by gene bootstrap approach as described by Falush *et al*. [44]. A consensus UPGMA tree based on the concatenated sequences was then stripped of branches which did not find 50% support in 1000 gene by gene bootstrap trees. The bootstrap approach identified 167 clusters of STs. Finally, we used BAPS on allelic identities with an upper bound of 400, which resulted in 216 clusters. For each of the three methods, many clusters each contained only one of the 138 eBGs and most or nearly all of the 138 eBGs contained isolates that were all assigned to a single cluster by each of the three alternative approaches (Table 1). The three methods were also largely congruent: for 108 eBGs, all the isolates were assigned to a single cluster by all three methods and for 24 others, the isolates were clustered together by two methods (Fig. 3). Finally, data permutation revealed that all of these correspondences between eBGs and the other methods were significantly non-random (*p*<10^−4^) except for the number of eBGs per BAPS cluster where 9.5% of the permutations contained at least as many single eBGs per cluster as were found with the unpermutated data (Table 1). We conclude that the large majority of our assignments of STs to eBGs reflects the existence of natural genetic groupings that can also be identified by multiple other, independent clustering algorithms. We also note that the analysis of 300 Kb from 114 isolates of subspecies *enterica* identified only four clusters other than Lineage 3, each containing isolates from one to three eBGs per cluster [45]. Thus, little phylogenetic information seems to exist above the cutoff imposed by our definition of an eBG, even when more extensive sequencing is applied.

**Table 1:** Comparison of groupings according to eBGs *versus* groupings by other algorithms.

	No. of	No. eBGs per cluster								Clusters per eBG		
Method	clusters	0	1	2	3	4	5	6	7	0	1	≥2
ClonalFrame	163	69	67	15	7	2	2	0	1	0	131	7
Bootstrap	167	33	132	1	0	0	1	0	0	14	112	12
BAPS	216	116	71	21	4	3	1	0	0	0	134	4

Note: Bootstrap is an abbreviation for the Gene by Gene Bootstrap approach with 50% support. 1092 distinct STs were tested by all three methods. BAPS with an upper bound of 400 assigned all STs to 216 clusters. The two other methods identified singletons (ClonalFrame, 189) or excluded individual STs whose branches did not receive 50% support (Bootstrap, 569), which were excluded from further comparisons. No. eBGs per cluster shows the numbers of clusters that contained 0, 1, 2,…7. eBGs according to each of the three methods. Clusters per eBG indicates the number of clusters identified by each of the three methods to which any STs within an eBG were assigned. Maximal number of clusters per eBG: BAPS, 2; ClonalFrame, 2; Bootstrap, 4. The significance of these associations was tested by 10,000 permutations of assignments of STs to eBGs for each of the three clustering assignments or by 10,000 permutations of the clustering assignments for the real eBG assignments of STs. None of the 10,000 permutations exceeded the number of eBGs found per cluster or the number of eBGs assigned to one cluster except that 9.5% of the permutations of the number of eBGs per BAPs cluster equalled or exceeded 71.

### Variable association between eBG and serovar

Some eBGs exhibit a unique one-to-one relationship with serovar, for example eBG13 (Typhi), eBG11 (Paratyphi A) and eBG26 (Heidelberg) (Table S1). Of the 48 eBGs containing at least 15 isolates, 22 contain a single serovar, or its monophasic variants. In contrast, 26 other eBGs contain multiple serovars (or isolates whose serovar is unknown), as indicated by white sectors in Fig. 2. Similarly, of the 42 serovars from which we sampled at least 15 isolates, 17 were associated with a single eBG but the remaining 25 serovars were associated with multiple eBGs and/or STs. Particularly dramatic examples of serovars that encompass multiple, distinct eBGs are Newport [12], Paratyphi B (see below) and Oranienburg (Fig. 2, Table S2) but multiple MLST clusters per serovar are common throughout the entire dataset, even in serovars from which only two isolates were tested (Fig. S2).

Discrepancies between serotyping and assignments to eBGs by MLST might reflect mistakes in serotyping or MLST sequencing, or both. Due to the decentralized sources of data, such mistakes almost certainly exist within the database. However, the MLST database is actively curated. Each nucleotide within a new MLST allele must be supported by at least two independent sequence traces before that allele is accepted by the curator, which has led to the rejection of multiple submissions of new alleles. All STs containing novel combinations of known alleles are examined visually for internally consistent genetic relationships to other STs and serovars. In multiple cases, this curation has resulted in rejecting such STs and subsequent resequencing of the gene fragments revealed technical errors. However, the most common discrepancy which we have encountered has been inaccurate serotyping, which has plagued several percent of database entries from all the laboratories involved in this project, as well as in ring trials for testing laboratory accuracy [52]. In numerous cases where the serovar and the ST of new entries were discordant with other isolates, re-serotyping revealed that the original culture had been contaminated, or had been inaccurately serotyped. However, despite active curation and rechecking serotypes and STs, multiple discrepancies remain between genetic relationships of STs and serovar, which are described below in greater detail for four test cases of increasing complexity.

### Serovar Typhimurium

**Figure 4:**
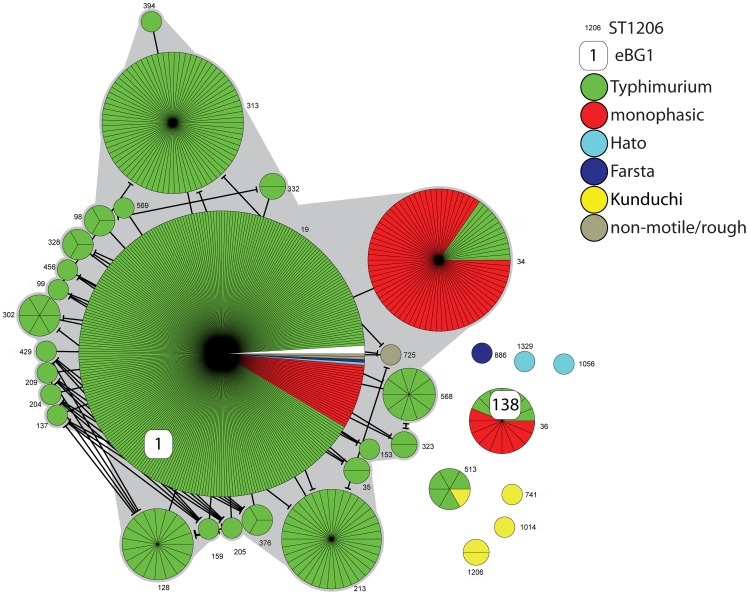
MSTree of Typhimurium plus its serological variants. Each circle represents one ST, subdivided into one sector per isolate, flanked by the ST number in small print. The primary links between STs within the MSTree are indicated by straight lines and additional cross-links at the same level of identity are indicated by lines that are terminated by bars. eBG designations are indicated by rounded white boxes. White sectors indicate a lack of serological information. Serological formulas are summarized in Table S3. Other details are as in Fig. 2.

eBG1 contained 482 isolates of serovar Typhimurium, which has the antigenic formula [1],4,[5],12:i:1,2 (Table S2). [The colons divide the epitopes within the lipopolysaccharide (LPS) O antigen (4,12) from those in the phase 1 flagellar antigen (i) and the phase 2 flagellar antigen (1,2). Numbers in square parentheses designate epitopes that are variably present within a serovar, in some cases due to lysogenic conversion by bacteriophages.] eBG1 also contained so-called monophasic variants of Typhimurium, 88 isolates that do not express the phase 2 antigen and four isolates that do not express the phase 1 antigen, as well as rough and non-motile variants (Fig. 4, Table S2). The presence of these serological variants within eBG1 indicates that they are genetically related to Typhimurium, and therefore these monophasic, rough and non-motile variants potentially represent mutations or recombination events affecting expression of LPS or the flagellar antigens encoded by *fliC* (phase 1) and *fljB* (phase 2). Prior work has indicated that monophasic variants represent multiple, independent genetic events [53], [54], and our results support this interpretation. ST19, the central ST in eBG1, contains two distinct forms of monophasic variants, and both monophasic as well as diphasic variants are also found in ST34. eBG1 also includes one isolate each of the serovars Hato and Farsta, whose antigenic formulas differ from Typhimurium at the phase 1 and 2 antigens, respectively (Table S3).

Not all Typhimurium isolates are grouped in eBG1 (Table S1, S3) and exceptional isolates were found in eBG138 and ST513. eBG138 shares only three identical alleles with eBG1 although it contains seven Typhimurium isolates plus nine monophasic Typhimurium isolates. Similarly, ST513 contains five Typhimurium isolates plus one Kunduchi isolate, whose phase 1 antigen differs from that of Typhimurium. ST513 also shares only three alleles with eBG1.

Thus, serotyping has conflated Typhimurium with isolates from genetically distant eBGs while failing to group related Typhimurium with its monophasic variants. Serotyping has also conflated genetically unrelated isolates of serovars Kunduchi, Farsta and Hato. Isolates of these serovars are found in six additional STs, each of which is unrelated to the others or to the STs containing Typhimurium (Fig. 4, Table S3).

### Serovars Enteritidis and Dublin

**Figure 5:**
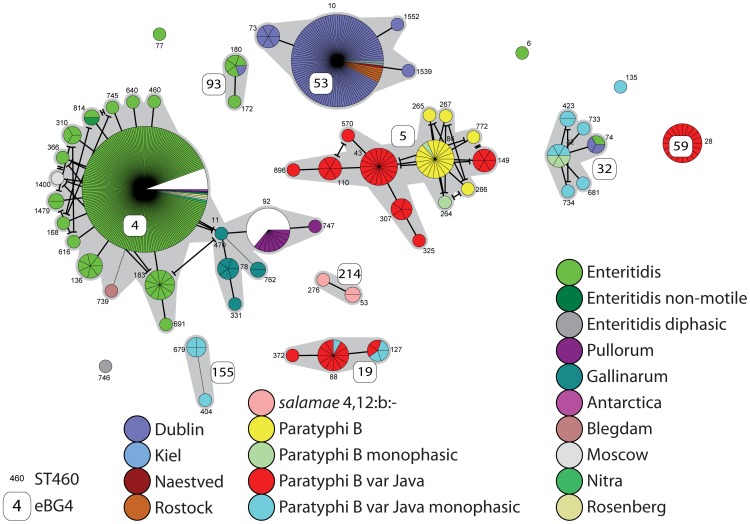
MSTree of Enteritidis, Dublin, Paratyphi B and their serological variants. Serological formulas are summarized in Tables S4 and S5. Other details are as in Fig. 4. Additional information on Paratyphi B and Java isolates can be found in Tables 2 and S6.

Two hundred and forty two serovar Enteritidis isolates ([1],9,12:g,m:-) were present in eBG4, as well as two non-motile variants (Table S2, Fig. 5). eBG4 also includes several serovars that differ from Enteritidis by their phase 1 (serovars Rosenberg, Moscow, Blegdam and Antarctica) or O antigens (Nitra) (Table S4). In addition, eBG4 includes a discrete sub-lineage consisting of multiple isolates of the serovars Gallinarum and Gallinarum var. Pullorum (henceforth referred to as Pullorum). In fact, Gallinarum and Pullorum are non-motile serological variants of Enteritidis that cause distinctive forms of lethal disease in poultry (fowl typhoid and pullorum disease, respectively), but can otherwise be difficult to distinguish because they differ in nutritional capabilities (biotypes) rather than serologically [55]. According to MLST, four STs containing Gallinarum were closely related to ST11, the most common ST in eBG4. Two STs containing Pullorum isolates branched from the basal Gallinarum ST, ST470 (Fig. 5). Similar results have previously been obtained with MLEE [56] and a genomic comparison of one strain each of Enteritidis and Gallinarum also indicated a close relationship [57]. Two Enteritidis isolates were assigned to ST77 and ST6, and a unique, diphasic Enteritidis isolate is in ST746, which are all unrelated to eBG4. Thus, like Typhimurium, most Enteritidis isolates are in one primary eBG but rare isolates are present in multiple unrelated eBGs and STs.

Serovar Dublin ([1],9,12,[Vi]:g,p:-) contains the flagellar p epitope rather than the m epitope in serovar Enteritidis. The majority (115) of Dublin isolates were grouped in eBG53, which shares only three alleles with eBG4, the main Enteritidis cluster, supporting this serological distinction. The remaining Dublin and Enteritidis isolates were found in eBG93 (Enteritidis: 5 isolates, Dublin: 1) and ST74 of eBG32 (Enteritidis: 1, Dublin: 1, Enteritidis/Dublin 1). eBG93 is intermediate between eBG4 and eBG53, sharing four alleles with each. ST74 shares none with either and other STs of eBG32 contained monophasic isolates of serovars Paratyphi B and Paratyphi B var. Java (henceforth Java) (Fig. 5), which only share the O12 antigen with Enteritidis or Dublin. It has previously been reported that strain RKS1550 (also designated SARB14; MLEE ET Du2) has the phase 1 antigenic formula g,m,p, which is a combination of the phase 1 antigens found in Enteritidis (g,m) and Dublin (g,p) [28]. Its FliC sequence encodes Ala220 and Thr315, which are typical of Enteritidis, as well as Ala318, which is typical of Dublin [28]. SARB14 was one of the three strains assigned to ST74. We confirmed by sequencing the presence of these three amino acids in its FliC sequence, and also found that the two other ST74 isolates possessed the same three substitutions. One of those two isolates had been serotyped as Dublin and the other as Enteritidis. However, we have now found that some such strains can be variably serotyped as Enteritidis, Dublin or both because different laboratories use different strains to generate and absorb serological typing sera.

In agreement with observations from MLEE [28], the primary Dublin eBG, eBG53, also includes six isolates of serovar Rostock. It also includes one isolate each of serovars Naestved and Kiel. Serovars Rostock and Naestved contain additional epitopes in the phase 1 antigen while serovar Kiel contains a distinct epitope in the O antigen. Rostock, Naestved and Kiel have not yet been found outside eBG53.

### Serovars Paratyphi B and Java

The observation that eBG32 contained Paratyphi B and Java isolates as well as Enteritidis and Dublin stimulated a closer examination of Paratyphi B and Java. The genetic relationships between Paratyphi B and Java have long been a topic of discussion. Their serological formula ([1],4,[5],12:b:1,2) is identical and Java is treated as a variant of Paratyphi B that can ferment d-tartrate (dTar+) whereas Paratyphi B *sensus stricto* is dTar- [58]. The dTar- phenotype has been attributed to a single nucleotide change in the start codon of the STM3356 gene, which is ATA in Paratyphi B rather than ATG [58], [59]. Paratyphi B is thought to be associated with typhoid-like fever in humans whereas Java is associated with non-invasive gastroenteritis in animals and humans [60], [61].

Our initial results did not allow a simple distinction between Paratyphi B and Java according to MLST, and these serovars seemed to be randomly distributed among various eBGs. After retesting all of the apparent exceptions plus numerous other isolates for their ability to ferment d-tartrate [58] as well as their phase 1 and phase 2 flagellar antigens, we found that the assignment to Paratyphi B or Java was inaccurate for 35/117 isolates, and that many Java isolates had been designated as Paratyphi B. Furthermore, many other isolates proved to be monophasic variants of Paratyphi B or Java (Table 2, S6). We also sequenced the start codon in STM3356 from numerous isolates. The results of these analyses showed that all Paratyphi B isolates with the ATA codon were in eBG5, within ST86 or five SLVs of ST86 (Fig. 5). Of these, ST86 and ST284 each contained one monophasic Paratyphi B isolate. However, three other monophasic Paratyphi B isolates were found in eBG32, although these had the ATG codon that has been associated with Java. Thus, it seems likely that classical Paratyphi B with an ATA codon arose once within eBG5 whereas an inability to ferment d-tartrate is associated with other genetic causes among monophasic Paratyphi B in eBG32.

**Table 2:** Comparison of assignments by MLST, MLEE, and molecular properties of Paratyphi B and Java isolates.

eBG	ST	#	MLEE^a^	Miko^b^	Prager^c^	Codon	Serovar
5	ST86	19	Pb1,Pb1a		SPV1	ATA	Paratyphi B + monophasic
5	ST43	18	Pb2	Group 1	EPV2	ATG	Java
5	ST110	6	Pb3, Pb3a		EPV2	ATG	Java
19	ST88	2	Pb4		EPV1	ATG	Java + monophasic
19	ST127	3			EPV4	ATG	monophasicJava
32	ST42, 423, 733, 734	11	Pb5, Pb5a, Pb5b, Pb5c		SPV2, EPV2	ATG	monophasic Paratyphi B + Java
59	ST28	21		Group 3, Group 2	EPV3	ATG	Java
	ST135	1			EPV4	ATG	Java
155	ST404, 679	5					monophasic Java
214	ST53, 276	3					*salamae*: 4,12:b:-

Note: #, Number of isolates in total. For detailed numbers in each category see Table S6. A mixture of Paratyphi B or Java with monophasic variants of the same serovar is indicated by ‘+ monophasic’.

a , Selander *et al*., 1990 [61].

b , Miko *et al*., 2002 [62].

c , Prager *et al*., 2003 [60].

Java was much more diverse than Paratyphi B (Fig. 5). Some diphasic Java were in STs of eBG5 other than those associated with Paratyphi B and others were in eBG19 and eBG59. Monophasic Java were found in eBG32 (together with the unusual monophasic Paratyphi B and Enteritidis/Dublin isolates described above) and in ST135. Monophasic Java isolates were also present in eBG19 and dTar^+^ isolates with the same antigenic formula were in eBG214, which is subspecies *salamae*. Taken together with a common inability to distinguish between Paratyphi B, Java and their monophasic variants, it is difficult to elucidate from the published literature just which eBGs are associated with typhoid-like fever and host specificity.

Our results were generally consistent with prior assignments of Paratyphi B/Java to distinct groupings by MLEE [61], molecular typing [62] and variable virulence determinants [60], suggesting that such groupings may correspond to individual eBGs and STs (Table 2, S6). However, even among the few isolates that were tested, we found multiple discrepancies between the different methods. Only 65/74 MLEE type Pb1 isolates were dTar- [61] *versus* 19/19 isolates within ST86. Virulence groupings SPV1, EPV1 and EPV3 [60] corresponded to ST86, ST88 and ST28, respectively, but EPV2 and EPV4 were each found in multiple eBGs.

These comparisons also revealed additional sub-differentiation within individual eBGs and STs. Virulence groupings SPV1 and EPV2, which differed in possession of SopE1 and frequency of SopD, correspond to distinct STs within eBG5, which indicates that virulence antigens need not be uniform within an eBG. Similarly, Miko *et al*. [62] reported that two distinct molecular groupings (Groups 2 and 3) of multidrug resistant (MDR) Java emerged in German poultry after 1994. Both groups were in ST28 of eBG59, showing that molecular fine typing can distinguish isolates within a single ST. Individual isolates within an ST can apparently also vary in regard to antibiotic resistance and its mechanisms because the Group 2 isolates possess a plasmid-borne class 1 integron whereas the Group 3 isolates contain a chromosomal Tn7-like class 2 integron [63]. Similarly, some MDR Java isolates from France carry the *Salmonella* genomic island 1 (SGI1), a ∼43-kb genomic island encoding multidrug resistance [64]. These isolates are in ST43 of eBG5, together with EPV-2 and Group 1, which do not contain SGI1 [63]. Thus, additional investigations are likely to reveal considerable diversity within eBGs and STs for virulence determinants and markers used for molecular typing.

### Population structure of 6,7:c:1,5 isolates

Typhimurium, Enteritidis, Dublin and Java are relatively common in Europe and the Americas, and have therefore been studied in considerable detail. In contrast, less information is available about subspecies *enterica* isolates with the antigenic formula 6,7:c:1,5, which have now become rare in Europe and the Americas. However, 6,7:c:1,5 isolates continue to be an important cause of invasive human disease in Asia and possibly elsewhere (Text S1). 6,7:c:1,5 isolates with apparently different disease specificities have been assigned distinct serovar designations on the basis of their differential abilities to ferment dulcitol and tartrate [2], [65] (Table 3), even though this distinction is based on biotyping rather than serotyping. Serovar Paratyphi C is associated with enteric fever in humans, Choleraesuis with septicemia in swine (and humans) and Typhisuis with chronic paratyphoid/caseous lymphadenitis in swine. Some Paratyphi C isolates express the Vi capsular antigen [66], which is otherwise associated with serovars Typhi and Dublin. Further biotypic subdivisions on the basis of H_2_S production and the utilization of mucate can be used to subdivide Choleraesuis into its variants *sensu stricto*, Kunzendorf [67] and Decatur [65] (Table 3), but these subdivisions are usually reached only by highly specialized reference laboratories. Earlier MLEE data showed that most Paratyphi C, Choleraesuis and Typhisuis isolates were genetically related, but others were distinct, including all of variant Decatur.

**Table 3:** Biotypes associated with serovars within 6,7:c:1,5 *S. enterica*.

Serovar	Dulcitol	Mucate	H_2_S	d-tartrate*
Decatur‡	variable§	+	+	ND
Paratyphi C	+	−	+	ND
Choleraesuis var. Kunzendorf	−	−	+	ND
Choleraesuis *sensu stricto*	−	−	−	+
Typhisuis	−	−	−	−

*: d- tartrate fermentation is only used to identify Typhisuis.

**‡:** Decatur was previously referred to as Choleraesuis var. Decatur [65].

**§:** Dulcitol is fermented by Decatur in eBGs 141 and 144 and STs 70 and 637 but not by eBG142.

The nutritional correlations between the different serovars are based on published information [2], [65] after modification due to the experiments described here.

**Figure 6:**
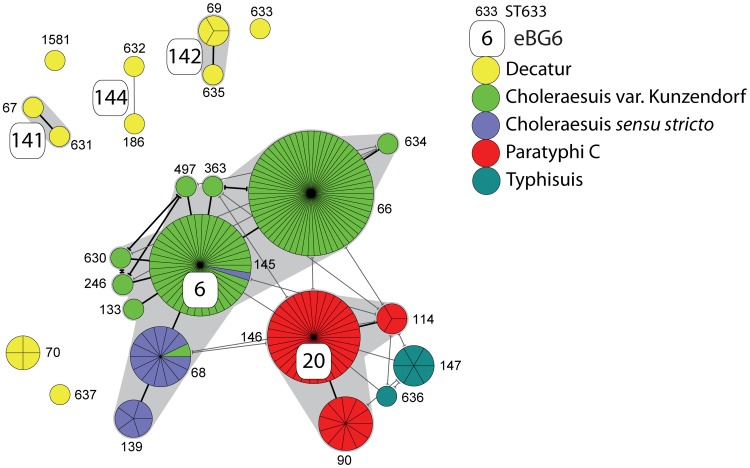
MSTree of 6,7:c:1,5 isolates. Details are as in Fig. 4 and additional information can be found in Tables 3 and S7.

We examined 202 supposed 6,7:c:1,5 strains isolated from animals and humans from global sources as well as from reference collections (Table S7). Most of these isolates had been assigned to Paratyphi C, Choleraesuis *sensu stricto* or Choleraesuis var. Kunzendorf, and we were only able to obtain eight supposed Choleraesuis var. Decatur and seven Typhisuis isolates. The collection included isolates from the SARB collection that represents the diversity of 6,7:c:1,5 isolates on the basis of MLEE [30]. A comparison of the nutritional characteristics of all these isolates with the MLST results resulted in the slightly revised differentiation scheme that is shown in Table 3. Our tests showed that 32 of the isolates had been serotyped incorrectly, or had not been assigned to the correct variant of Choleraesuis, including exceptional isolates according to MLEE. Two others were not even 6,7:c:1,5. After correcting these faulty serovar assignments (Table S7), both MLEE and MLST assigned all Choleraesuis, Paratyphi C and Typhisuis isolates into a single complex of genetically related eBGs and STs that are subdivided by serovar (Fig. 6).

All 48 Paratyphi C isolates were assigned to three STs within eBG20. Early in the 20^th^ century, microbiologists subdivided Paratyphi C into varieties Orientalis and Hirschfeld on the basis of their geographical site of isolation. The three isolates with such designations were in ST90, which also contained standard Paratyphi C. When tested by a PCR assay for multiple genes within the SPI7 genomic island that encodes the Vi antigen, all Paratyphi C isolates that were tested were positive for the entire SPI7 island, or for a modified version designated ΔSPI7 because it contains an internal 5 kb deletion. All other 6,7:c:1,5 isolates tested were negative (Table S7). Typhisuis isolates were assigned to ST147 and ST636, which differ by two alleles from each other and from the central ST of eBG20.

All 128 Choleraesuis isolates were grouped in eBG6, which is a DLV of eBG20 (Fig. 6). Within eBG6, two related STs are largely composed of Choleraesuis *sensu stricto* isolates, which do not produce H_2_S, whereas the other eight STs are largely composed of Choleraesuis var Kunzendorf, which do produce H_2_S. Paratyphi C isolates also produce H_2_S, suggesting that var Kunzendorf might have been ancestral and *sensu stricto* (STs 68 and 139) corresponds to a lineage that has lost the ability to form H_2_S. The association between H_2_S production and ST is not absolute because one exceptional var Kunzendorf isolate was found in a *sensu stricto* ST and one *sensu stricto* isolate in a Kunzendorf ST.

Other 6,7:c:1,5 isolates were unrelated to the complex consisting of eBG6, eBG20 and Typhisuis. These isolates included strain SARB5 (MLEE electrophoretic types Cs6) and SARB7 (Cs13). Published data [68] as well as our biotyping indicate that SARB5 (Cs6) is a Choleraesuis var. Decatur, and MLST assigned it to eBG141 together with a second Decatur isolate (Fig. 6). Similarly, SARB7 (Cs13) is Dulcitol-negative, H_2_S-positive, Mucate-positive, and Tartrate-positive, which we now also score as Choleraesuis var. Decatur (Table 3). SARB7 was assigned to eBG142 by MLST together with three other strains of the same biotype that were isolated in the same country (Australia) and year (1988). Similarly, MLEE ET Ts3 (SARB70) was supposed to be a Typhisuis isolate that was more closely related to Decatur than to other Typhisuis strains [30]. Once again, SARB70 is Choleraesuis var. Decatur, according to published data [30] plus our own biotyping results. SARB70 was assigned to ST70 by MLST along with SARB8 and two other Decatur isolates, one of which had originally been typed as Typhisuis var. Volsdagsen. Thus, Decatur consists of at least seven lineages (eBG141, eBG142, eBG144, ST70, ST633, ST637 and ST1581), which are only distantly related to each other or to the main group of 6,7:c:1,5 isolates described above. These observations argue against the current concept that Decatur is a variant of Choleraesuis and also argue against assigning any common designation to them despite their similar biotypes.

### Sequences of flagellar antigens

**Figure 7:**
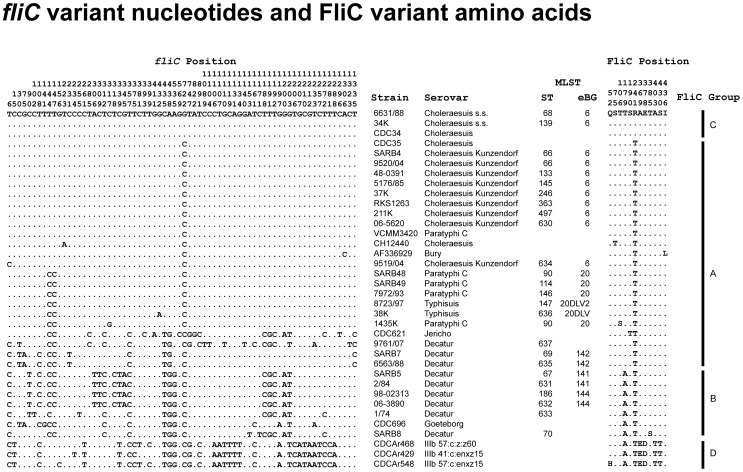
Variant nucleotides in the *fliC* gene and variant amino acids in the FliC protein. Sequences of *fliC* (Table S8) from isolates investigated here and additional sequences from GenBank with ≥97% BLAST identity and ≥97% coverage were trimmed to a length of 1344 bp, beginning at nucleotide 73 of the *fliC* gene of strain LT-2. The translated 448 amino acid sequences begin at amino acid 24. The figure shows all differences relative to the uppermost sequence (strain 6631/88), with nucleotide differences at the left and amino acid differences at the right. FliC protein groups were assigned with the help of a UPGMA tree (Fig. S4) and are indicated at the far right. Strain and serovar designations are in the center, followed by MLST ST and eBG designations for the strains investigated here.

If Decatur are both diverse and genetically distinct from standard Choleraesuis, Paratyphi C and Typhisuis, why do they all share the same serotyping antigens? To address this question, we sequenced almost all (1300/1500 bp) of each of the phase 1 *fliC* and phase 2 *fljB* genes of representative isolates from various STs (Table S8). BLAST searches against GenBank with representative sequences from Paratyphi C or Choleraesuis isolates identified additional nearly identical sequences (*fliC*: ≥97% identity, ≥97% coverage; *fljB*: ≥95% identity, 100% coverage), which were also included in the analyses. For *fliC*, strong BLAST hits were found not only among Choleraesuis and Paratyphi C isolates but also among other isolates that express the phase 1 c epitope in serovars Bury, Jericho, Goeteborg as well as in subspecies IIIb (*diarizonae*) (Fig. 7). Only a limited number of nucleotides were polymorphic in these sequences, and most of those polymorphisms were synonymous and did not introduce any amino acid changes. As a result, a total of only 12 amino acids were polymorphic in the FliC protein sequences, which subdivided the sequence variants into four slightly distinct groups (Fig. S4). Most of the polymorphic amino acids were associated with subspecies IIIb isolates, but five were polymorphic among Choleraesuis, Paratyphi C, Typhisuis and Decatur (Fig. 7). These polymorphisms were not uniquely associated with any serovar, nor did any single amino acid reliably distinguish Decatur from the main 6,7:c:1,5 groups. Choleraesuis *s.s.* was in FliC group C, Choleraesuis var Kunzendorf, Paratyphi C, Typhisuis, some Decaturs, Bury and Jericho were in FliC group A, and other Decaturs and Goeteborg were in FliC group B. The near identity of the FliC sequences from the genetically distinct isolates in various serovars likely reflect horizontal gene transfer by homologous recombination between these lineages.

**Figure 8:**
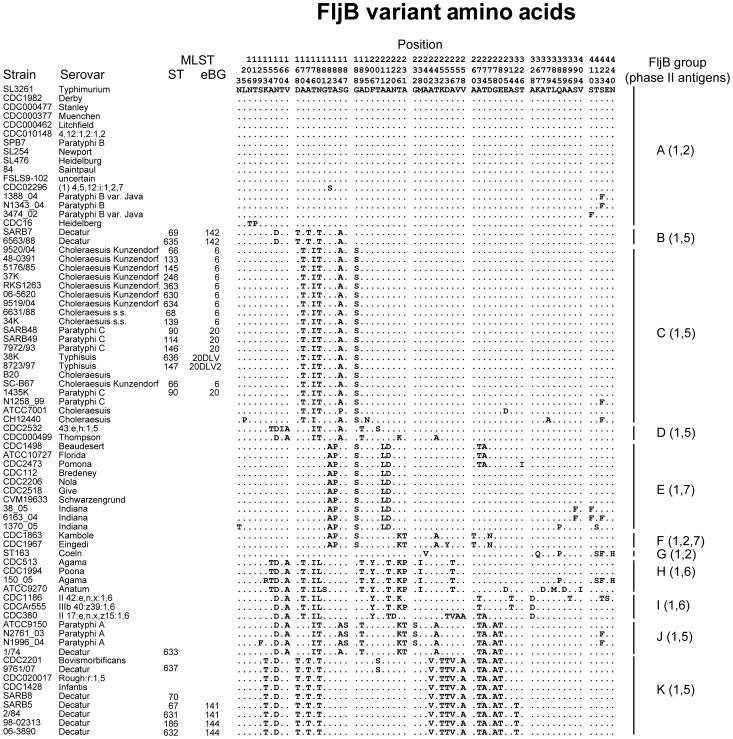
Variant amino acids in the FljB protein. Sequences of FljB (Table S8) from isolates investigated here and additional sequences from GenBank with ≥95% BLAST identity and 100% coverage were trimmed to a length of 440 amino acids, beginning at amino acid 36 in the FljB protein from strain LT-2. The figure shows all differences relative to the uppermost sequence (strain SL3261). FljB protein groups were assigned with the help of a UPGMA tree (Fig. S7) and are indicated at the right, together with the serological factors in the phase 2 flagellar antigen. Strain and serovar designations are at the left, followed by MLST ST and eBG designations for the strains investigated here.

Greater diversity was observed for *fljB*, resulting in assignments to 11 amino acid groups, A through K (Fig. 8, supplementary Figures S5–S7). This greater diversity arose in part because the BLAST searches had identified strongly homologous sequences expressing FljB epitopes 2, 5, 6, or 7 in combination with epitope 1. These have previously been referred to as the 1-Complex [14]. The 11 amino acid groups correlated in large part with the FljB serological epitopes, e.g. group A sequences were 1,2 while B sequences were 1,5. However, multiple sequence clusters were found for each set of epitopes, e.g.1,2 was associated with groups A, F and G and 1,5 was associated with groups B, C, D, J and K. The sequence differences between groups expressing the same serological epitopes were in part as large as the differences between distinct sets of epitopes. Genetically distinct eBGs and STs tended to belong to distinct FljB sequence groups: group B included the Australian Decatur isolates in eBG142; group C encompassed the related Choleraesuis, Paratyphi C and Typhisuis isolates; and group K encompassed the other Decatur isolates. Thus, it might be possible to develop molecular serotyping tools that could distinguish some of these distinct eBGs and STs. However, more efficient serology or molecular serology would not distinguish between eBGs 6 and 20 or eBGs 141 and 144, because each of these paired groups contained identical FljB sequences.

These results show that classical serotyping has been very efficient at recognizing identical or closely related sequences of FliC. It has been less efficient at distinguishing distinct sequences of FljB that differentiate Decatur and the Choleraesuis/Paratyphi C group, which has resulted in serological conflation of these genetically unrelated serovars.

We were intrigued by the apparent rarity of non-synonymous polymorphisms, particularly in *fliC*. We therefore compared ω, the relative frequency of non-synonymous polymorphisms to synonymous polymorphisms in *fliC* and *fljB*, with ω in the individual MLST genes (Table 4). These results show that neither *fliC* nor *fljB* is particularly unusual, because *d*N, *d*S and ω are within the range found for MLST genes. A relative lack of non-synonymous polymorphisms within housekeeping genes is generally attributed to purifying selection due to the loss of deleterious mutations that led to amino acid changes. Given the similar values for ω in *fliC* and *fljB*, purifying selection should be considered as the null hypothesis for the relative absence of non-synonymous polymorphisms as well.

**Table 4:** Relative frequencies of synonymous and non-synonymous polymorphisms.

Gene	Alleles	*d*N	*d*S	ω
*purE*	278	0.0050	0.076	0.067
*aroC*	274	0.0015	0.038	0.041
*dnaN*	287	0.0011	0.047	0.024
*hemD*	196	0.0076	0.032	0.241
*hisD*	338	0.0027	0.064	0.042
*sucA*	248	0.0007	0.045	0.016
*thrA*	292	0.0007	0.054	0.014
*fliC*	36	0.0016	0.031	0.052
*fljB* (1,5)	38	0.0040	0.018	0.220
*fljB* (all)	73	0.0140	0.057	0.248

Note: Alleles, number of unique sequences. *d*N, average frequency of non-synonymous mutations per potential non-synonymous site. *d*S, average frequency of synonymous mutations per potential synonymous site. ω, *d*N/*d*S. *fljB* (1,5), only sequences from FljB amino acid groups A, B and C. *fljB* (all), all unique sequences in Fig. 8.

## Discussion

The data and analyses presented here provide a broad overview of the population structure of *S. enterica* subspecies *enterica* using a bottom-up approach. Taxa can be subdivided by a top-down approach, phylogenetics, which elucidates a genealogical tree, or a bottom up analysis, population genetics, which identifies populations and/or networks. Trees are appropriate for clonal organisms with an unambiguous genealogy, such as *Y. pestis* [69] or serovar Typhi [11], whereas the identification of populations is more appropriate for organisms which experience frequent homologous recombination, such as *Helicobacter pylori* [70]. Some bacterial taxa can be successfully analyzed by a combination of both approaches (e.g. [71], [72], and new approaches are being developed that explicitly include lateral gene transfer events as part of the genealogy [73]. However, classical phylogenetic approaches to elucidate the genealogy of *S. enterica* from MLEE data [29], [31], [61] or genomic sequences [48] are potentially falsified by frequent recombination [7] and therefore difficult to interpret. In contrast, the bottom-up definition of eBurst groups on the basis of allelic identity rather than sequence identity [46], [47] tends to yield discrete clusters of related organisms even at medium levels of homologous recombination [43].

The population structure of subspecies *enterica* consists of numerous, discrete starburst-like clusters of STs (Fig. 2) that we designate as eBGs. The existence of these clusters is visually obvious within a minimal spanning tree (Fig. 2) and our assignments to eBGs are strongly supported by three additional, independent methods based on sequence homology or allelic identity (Fig. 3). Our definition of eBGs raises multiple questions. Will these assignments remain stable as 10,000 s of additional isolates are investigated by MLST and genomic analyses? What are the evolutionary mechanisms that have resulted in discrete genetic clusters? And what are the predictive properties of co-membership within an eBG?

### Future stability of eBG assignments

The definition of eBGs is based on longer branches between eBGs than within eBGs. Active curation of all new STs will help to prevent filling of such gaps through error, such as the artificial creation of mosaic STs due to mixed cultures or sequencing reactions. However, it might be expected that with time those gaps will be closed through the identification of rare, intermediate STs, such as result from homologous recombination. Indeed, the merging of clusters through recombination is predicted by simulations [43] and has been observed within many species, such as *E. coli* [7], *Y. pseudotuberculosis* [74] or *Campylobacter jejuni* [75]. Within subspecies *enterica*, intermediate STs between eBGs of serovar Newport have also been attributed to homologous recombination [12]. In such cases, we recommend following the practise implemented for MLST of other pathogens such as *Neisseria meningitidis*, where eBurst group designations are maintained for groupings with distinct epidemiological patterns even when these groups become linked by rare, novel isolates.

We anticipate a potential problem with eBGs within Lineage 3 (Fig. S2) because only limited numbers of Lineage 3 isolates have been investigated and recombination among those isolates is particularly frequent [45]. Lineage 3 may in fact represent a connected network rather than multiple independent starbursts. In that case, eBGs within Lineage 3 might need to be merged into larger eBGs with time, as has occurred for particular lineages within *N. meningitidis* and *E. coli*, or the use of eBG designations might need to be discarded for Lineage 3. However, we expect that most eBGs outside of Lineage 3 will continue to exist even after 10,000's of additional strains and genomic sequences have been obtained.

Our optimism about the durability of most eBGs is based on the strong correlations between serotyping and eBG assignments for multiple eBGs, as well as our general failure to identify intermediate STs after extensive searches. For example, we were struck by the distinctiveness of eBG13 (Typhi) [10], [11] and attempted to identify related STs by examining rare serovars with overlapping antigens. 100,000 s of isolates from subspecies *enterica* have been serotyped and 1500 serovars have been defined. Yet none of the rare isolates with overlapping serotypes were genetically related to eBG13 according to MLST (data not shown). Similarly, we investigated 200 6,7:c:1,5 isolates from global sources, but failed to identify any ST that joined eBGs 6 (Choleraesuis) and 20 (Paratyphi C). Our unpublished genomic analyses of serovars Paratyphi A and Agona confirm that each of these serovars represents a tight genetic grouping without close relatives. Thus, although we are somewhat uncertain about the durability of eBGs within Lineage 3, we are confident that most eBGs represent natural groupings that will not be demolished by additional data.

We also anticipate that some higher order relationships between eBGs may be detected by genomic analyses. For example, our distinction between eBG6 and eBG20 is based on a difference of two of the seven alleles between the most closely related pairs of STs within these eBGs. It maintains microbiological tradition and reflects distinctive disease and host properties. In contrast, eBG6 and eBG20 were clustered together in a higher order evolutionary grouping, Lineage 1, according to analyses of multiple gene fragments spanning 300 kb [45], and they also cluster together within the MSTree. Such higher order groupings may reveal details about longer term evolutionary history but do not invalidate the lower level clustering represented by eBGs.

### Evolutionary sources of eBGs and predictive properties

We conclude that eBGs represent natural groupings, but are uncertain about why they exist, how they arose and what can be predicted from an assignment to an eBG. Clearly, eBGs represent groups of closely related organisms related by descent from a common ancestor. However, the time scale of that descent is uncertain, within subspecies *enterica* as well as almost all other bacterial pathogens, because the mutational clock rate can vary by orders of magnitude between bacterial taxa [76]. It is tempting to equate eBGs with ecotypes, relatively uniform clusters of organisms sharing a common ecological niche which are continuously purified of diversity via competition and selective sweeps [77]. However, the utility of the ecotype concept is controversial for pathogens [78], and even for environmental organisms [79]: Neutral processes such as bottlenecks and changes in population size can lead to reductions in diversity even in the apparent absence of selective sweeps [69] and uniformity does not necessarily reflect population wide replacement by a fitter variant because selection can be at the level of individual genes or gene clusters [79]. Thus, the evolutionary pressures leading to eBGs are currently best regarded as an interesting topic which warrants further investigations of evolutionary and population genetic history through genomic sequencing of defined collections.

The predictive properties of eBGs are similar to those of serovars, some of which are thought to have undergone host-adaptation due to specific associations with host and disease type [80]. For example, serovars Typhi, Paratyphi A and Paratyphi C all cause typhoid or enteric fever (exclusively) in humans, and each belongs to a distinctive eBG. And even though they are genetically closely related, eBGs distinguish between Choleraesuis (eBG20), Paratyphi C (eBG20) and Typhisuis, which differ in host adaptation: Choleraesuis can infect multiple mammalian species and causes a different form of invasive disease in humans than does Paratyphi C [81]. However, sufficient numbers of discrepancies exist between serovars and eBGs that the question of host-adaptation needs to be revisited for multiple eBGs. For example, Choleraesuis var. Decatur consists of multiple, genetically unrelated eBGs, each of which is also distinct from Choleraesuis. Paratyphi B var. Java and its monophasic variants are also distributed across multiple eBGs. Varying disease potential (if any) of these different eBGs will first become apparent after analyses of the correlations between disease state with eBG, which has not yet been performed. In some cases, serotyping may be more predictive of host-adaptation, e.g. Paratyphi B isolates form a sub-cluster within eBG5, which otherwise contains Java isolates whose disease potential is uncertain. Similarly, serovars Gallinarum and Pullorum, which cause fowl typhoid and pullorum disease, are grouped within one sub-cluster of eBG4. The other primary sub-cluster in eBG4 consists of serovar Enteritidis, which can cause a variety of other diseases in multiple hosts. Other observations also suggest occasional host-specificity at the ST level rather than the eBG level. ST183 in eBG4 (Enteritidis) contains phage type 11isolates from hedgehogs in Germany and humans in the UK. In eBG1 (Typhimurium), phage type DT56var isolates from finches and humans in the UK were in ST568 [82] and phage type DT2 isolates from pigeons in Germany and France were in ST128.

### Serovars and their problems

The assignment of isolates to serovars on the basis of serotyping plus nutritional characteristics, the Kauffmann-White scheme, was initiated over 70 years ago, with the deliberate intention of providing a scheme with limited resolution that could be implemented in multiple laboratories [83]. Serovars were never intended to permit the complete differentiation of all antigenic diversity, nor was the serotyping scheme ever claimed to be complete or final [84]. Serovar designations continue to be updated regularly as new insights are acquired [2], and some of the discrepancies between eBGs and serotyping have resulted in new serovar designations (Table S1, S2) that will be implemented in the next version of the scheme.

The serovar concept is practised globally, providing a universal language of communication. 100,000's of isolates are serotyped annually and serovars are the basis for public health measures to reduce zoonotic diseases. However, in the interests of correctly identifying potential causes of disease with greater accuracy and higher speed, we recommend phasing out the routine use of serovars, and replacing it with a classification that is based on population genetic groupings such as eBGs and STs. This recommendation derives from the existence of multiple problems with assignments to serovars. Serotyping has multiple technical disadvantages, including low throughput, high expense, as well as a requirement for numerous antibodies made by immunizing rabbits plus considerable expertise [13], [14]. Serotyping remains error-prone, even for the most common serovars, as demonstrated repeatedly here and in small scale ring trials [52], and is not amenable to automation. However, our primary criticism of *Salmonella* serotyping reflects its information content. Some serovars are genetically relatively homogeneous, e.g. Typhimurium or Enteritidis, and most isolates from such serovars are closely related and belong to a common eBG. In contrast, numerous other serovars were distributed across multiple eBGs and/or STs (Fig. 2), and are therefore not necessarily uniform in virulence or epidemiology. Thus, serovars conflate eBGs with different biological properties, e.g. Decatur and Choleraesuis. For serovars such as Kentucky, Newport, and Java, it is not even possible to define a primary eBG because numerous isolates of those serovars were found in multiple distantly related groups (Table S2). At the same time, serovars differentiate between individual isolates that are closely related genetically but happen to possess distinct lipopolysaccharide or flagellar epitopes due to horizontal gene transfer or mutation, e.g. Dublin and Rostock, or Typhimurium and Farsta: 26 of the 48 eBGs containing at least 15 isolates included two or more serovars. Our results also show that serotyping is inconsistent. eBG1 contains monophasic variants that cannot be assigned a serovar designation because their epitopes are not unique whereas Java encompasses both diphasic and monophasic variants as well as multiple eBGs. And the assignment of an isolate to a serovar is often dependent not only on serology but also on nutritional properties, such as the differentiation between Choleraesuis, Paratyphi C and Typhisuis. We have primarily focused on well known serovars here because they represented the largest number of isolates that were tested by MLST. However, polyphyletic serovars are common, even those that are isolated only rarely in the USA or Europe (Fig. S8).

Possibly the strongest arguments for continuing to assign isolates of *S. enterica* to serovars are tradition, the extensive infrastructure for serotyping in public health laboratories, and familiarity. Although it is difficult to discard a system that has been used so extensively for >70 years, and which is so embedded in microbiological thinking, the use of serotyping alone is often uninformative. Most of the *S. enterica* isolates in any European country belong to a very limited number of serovars, usually fewer than 10 (Fig. S8). In fact in recent years, most isolates belonged to Enteritidis, Typhimurium or Infantis, which results in relatively low discrimination. Furthermore, many current isolates of Typhimurium are monophasic and cannot be unambiguously recognized by serotyping [85]. Epidemiological investigations of outbreaks often depend on phage typing [86], PFGE [17], [18] or MLVA [19], alone or in combination, usually after initial triage based on serotyping. These methods could continue to be used, and are likely to be even more effective if combined with an initial assignment to genetic groupings such as eBGs.

### MLST for *S. enterica*

MLST was first described in 1998 [87] and has now become the gold standard for long term epidemiology and population genetic analyses of pathogenic microbes. Of the 79 MLST databases that are publicly available (http://pubmlst.org/databases.shtml), the *S. enterica* MLST database (http://mlst.ucc.ie) ranks fourth in number of isolates. This publicly accessible and actively curated web-based MLST database facilitates the global exchange of information. In particular, new alleles and new STs depend on user submissions rather than decisions by a central reference laboratory, and are immediately made publicly accessible. Similar global exchange of information at the strain level does not exist for serotyping. The database currently provides data for >500 of the 1,500 existing serovars in subspecies *enterica*, including all common serovars and many that are rare. These data have been accumulated through a decentralized global effort since 2002 and with time, we anticipate that representatives of all 1,500 serovars will be tested, thus providing a reasonably complete mapping between serovar and eBG/ST.

The data presented here demonstrate that MLST is a valuable tool for the identification of genetic clusters and elucidating the diversity of known serovars. We also believe that it has the potential to completely replace serotyping, over which it possesses multiple advantages. Replacement of serotyping by MLST would involve changes in nomenclature. In cases where eBGs are relatively uniform in serovar and correspond to monophyletic groups, the serovar designations could be maintained together with the eBG designation for an interim period in order to provide continuity, e.g. eBG1 (Typhimurium). For polyphyletic serovars, the serovar designation has little information content and should be eliminated as soon as possible, as is the case for other species for which MLST has become the common language. Even now, a surprisingly large numbers of entries are already being deposited at the MLST website without accompanying serovar information.

In private discussions, some individuals have claimed that MLST is too technically demanding, expensive and slow. However, performing MLST does not require much more than a PCR machine plus training on working with DNA sequences. Our experience is that MLST does not require much technical competence, and laboratory scientists who are capable of handling serotyping can readily learn to handle MLST. MLST is cheaper than serotyping, sequencing of PCR products can be performed commercially and it can be automated. In our hands, with the help of robotic fluidics, one individual can easily complete the necessary manipulations from initial single colony isolation through to finished sequencing at the rate of 200 isolates per week and a cost per isolate of under €25. A few days are needed to enter the sequence traces into a database and evaluate them with the help of dedicated scripts. In general, a small fraction of traces need to be repeated, which then doubles the time needed to provide definitive results for all 200 isolates. We anticipate that in the future, technical developments will allow even higher throughput of MLST assignments through multiplexed SNP-based typing and/or next-generation sequencing.

Other individuals have claimed that MLST will soon be replaced by whole genome sequencing (WGS), whose price is rapidly approaching that of MLST. Instead we argue that WGS and MLST are complementary, and should be pursued in parallel. WGS provides essential information for epidemiological tracking and will yield invaluable insights into the detailed population structure of bacterial pathogens [69], [88], including *S. enterica*. However, the evaluation of SNPs and genomic sequences from WGS takes much more time than the evaluation of paired traces from seven gene fragments. WGS currently suffers from differences between samples in quality and number of reads per nucleotide, which presents difficulties in extracting identical gene fragments from multiple genomes due to variable missing data. The *S. enterica* MLST database will probably contain data for >10,000 isolates in the near future, as do three other MLST databases today, whereas it would currently be difficult to extract information with comparable certainty from that many genomes. We propose that MLST should be used to provide a rapid overview of the population structure of *S. enterica*, which can then be used to identify selected isolates for investigation in greater detail by genome sequencing. Such efforts including the integration of genomic sequences and MLST data are already underway [89].

A third criticism of MLST for *S. enterica* is that it does not provide the fine resolution needed for outbreak analysis and short-term epidemiology. Indeed, MLST data does not generally have the same fine resolution as phage typing, PFGE, and MLVA. Multiple phage types were present within ST19, the central ST in eBG1 (Typhimurium), and within ST11, the central ST of eBG4 (Enteritidis, Gallinarum, Pullorum). However, MLST does provide somewhat greater resolution than serotyping because eBGs tends to contain multiple STs once a sufficient number of isolates has been tested. On occasion, MLST has also given hints of phylogeographic and host specificity. For example, invasive disease caused by Typhimurium in Africa is associated with ST313 and its descendent SLVs within eBG1 [39]. ST213 within eBG1 has only been isolated in Mexico [38]. Similarly, STs 66 and 634 of eBG6 (Choleraesuis) were first isolated in Canada (1978) and the USA (1981–1986) and subsequently from humans and swine in Taiwan (1998–2004). A potential link between these isolates may have been breeding pigs, which have been imported into Taiwan from Canada and the USA since 1980 (http://www.angrin.tlri.gov.tw/indexd/AGLP.htm).

We conclude that MLST is a powerful candidate for the reference classification system for *Salmonella*, and can replace serotyping for that purpose. Similar to serotyping, additional methods will be needed to provide the fine resolution that is required for short term epidemiology. In other species where serotyping was previously the common language for strain tracking and epidemiology, such as *E. coli* or *Klebsiella pneumoniae*, it was rapidly replaced by MLST nomenclature after its introduction. We are confident that MLST designations will be also be adopted widely in the near future for *S. enterica*. By eliminating multiple misleading interpretations about strain relatedness associated with serotyping, this step would represent a major improvement for the epidemiology and control of *Salmonella* infections.

## Materials and Methods

### Bacterial strain collection and microbiological properties

The analyses presented here are based on 4257 isolates whose data has been submitted to http://mlst.ucc.ie/mlst/dbs/Senterica by ourselves and others. Of these, 1770 are maintained in the strain collection of MA at University College Cork, and 1042 in the strain collection of FXW at the Institut Pasteur, for a total of 2643 in either or both of those collections. Biotyping and serotyping were performed in multiple laboratories but most of the tests were performed under the supervision of FXW or MC. Serotyping and biotyping were according to the modified Kauffmann-White scheme [2], except as described below.

Basic information on all isolates can be downloaded from the website. In addition, a detailed description of strain properties for Paratyphi B and Java isolates is presented in Table S6. The distinction between Paratyphi B and Java was based on two tests, which gave concordant results after up to 7 days incubation: the lead acetate protocol 1 for d-tartrate fermentation described by Malorny *et al*. [58] and the ability to grow on d-tartrate as the sole carbon source as described by Weill *et al*. [64]. The start codon of STM3356 was sequenced as described by Malorny *et al*. [58].

Table S7 gives detailed information on results with 6,7:c:1,5 isolates. These were assigned to serovars on the basis of the biochemical properties which are summarized in Table 3, and which are similar to the tests and recommendations by Le Minor *et al*. [65]. Mucate utilization, ducitol fermentation and H_2_S production were evaluated after 24 hrs incubation in standard media and tartrate fermentation was evaluated after 7 days, as described above.

A separate manuscript is in preparation on differences between the contents of Selander's SARA and SARB collections. The conclusions drawn here were largely based on isolates stored by Kenneth E. Sanderson and corroborated by the collection of Fidelma Boyd. Serovar assignments were according to information uploaded to the website except that many atypical isolates and the Paratyphi B, Java and 6,7:c:1,5 isolates were retyped.

### DNA sequencing

MLST was performed on seven gene fragments as described [9], [12] using the amplification and sequencing primers that are described on the MLST website. Sequences for each gene fragment were assembled from at least two independent PCR products, and trimmed to a constant length of 399–501 bp as indicated on the website. All allelic sequences and allelic combinations can be freely downloaded from the website.

*fliC* and *fljB* were sequenced using the same oligonucleotide primers for PCR amplification and sequencing as previously described [90], [91]. These primers each yield a ∼1500 bp product, which were trimmed to correspond to positions 73–1344 within the *fliC* gene and 109–1428 within the *fljB* gene, as shown in Figs. 6 and S5. Sequences have been deposited in GenBank under the accession codes HQ871156–HQ871237 (Table S8).

### Microarray analysis of SPI-7 (Salmonella Pathogenicity Island-7)

A custom oligonucleotide probe-based array was designed as previously described [92] in order to detect genes related to the absence and presence of SPI-7. After labelling, probes were purified and applied to microarray slides [93]. Genomic DNA was sonicated to yield 200–500 bp fragments, purified and labelled with Cy3-dCTP using the BioPrime DNA Labelling System (Invitrogen–BioSciences Ltd., Dun Laoghaire, Ireland). Duplicate slides were hybridized with the dCTP labelled DNAs in 48% formamide at 55oC for 16–20 hrs in a humid chamber. The slides were washed at RT, washed again at 50oC, scanned (GenepixR 4000B laser scanner, Axon Instruments, Redwood City, Calif.) and processed (GenePix Pro 3.0). The full dataset was analyzed using R (www.r-project.org), and Bioconductor (www.bioconductor.org) as described [94]. In brief, the bimodal distribution that was observed was treated as two overlapping Normal distributions. Means and 95% confidence intervals were determined for each distribution. Probes were scored “absent” if the log2 intensity was within or below the 95% CI for the “low” peak; “present” if the log2 intensity was within or above the 95% CI for the “high” peak and intermediate values were scored as “uncertain”. As a control, PCR tests similar to those described previously [95] were used to screen for presence or absence of larger regions of SPI-7.

### Phylogenetic analyses

Concatenated sequences from all seven gene fragments within 1092 STs were aligned using Mega 4 [96] and analyzed by ClonalFrame [51], yielding the tree in Fig. S3 and a total of 903 clustered STs in 163 groups. Gene by gene bootstraps [44] were also performed on 1092 STs, except that for each of 1000 iterations, the seven gene fragments used for concatenation were chosen at random from the seven genes, with replacement. UPGMA trees were generated from all 1000 iterations using Paup [97] and a homemade script in Perl (available on request) was used to generate a 50% consensus tree based on the percentage support for each branch. 569 branches to individual STs that did not meet these criteria were excluded by this script. dN and dS were calculated on each gene fragment using Mega. UPGMA trees of the fliC and fljB nucleotide sequences and the FliC and FljB amino acid sequences were generated in Bionumerics 6.5 (Applied Maths, Sint-Martens-Latem, Belgium), as shown in Figs. 7–8 and S4–S7. Maximum likelihood topologies of synonymous and non-synonymous sites were calculated using PhyML [98].

### Clustering analyses

A minimal spanning tree was generated from the allelic profiles of isolates using the predefined template in BioNumerics 6.5 designated as MST for categorical data, which preferentially joins single and double locus variants with the largest number of isolates per ST. For allelic comparisons, Baps 5.3 [49] was applied to the allelic profiles from each ST with an upper bound for group numbers ranging between 300 and 500. The number of clusters ranged from 215 to 221 as the upper bound increased. The data presented here are based on an upper bound of 400, which yielded 216 clusters. Baps was also used with allelic differences with an upper bound of 2–7 or with concatenated sequences (Fig. S2) as described in Text S1.

## Supporting Information

Figure S1 MSTree from Fig. 2 color-coded according to BAPS assignments to five clusters of allelic differences among 1097 STs. STs assigned to lineage 3 are colored in red and the four other colors indicate four other clusters of STs. Similar results were obtained with BAPS or STRUCTURE assignments to 5 clusters based on concatenated sequences of the seven MLST genes. The existence of STs from the other four clusters near the bottom of the figure is due to rare intermediate STs with recombinant alleles that artificially join lineage 3 to other clusters in a minimal spanning tree.
(PDF)

Figure S2 *H*, the index of genetic diversity, versus number of isolates per serovar in the MLST database. *H* was calculated as (*n*/(*n*-1))*(1.0 - the sum of squares of the relative frequency per serovar of isolates in discrete eBGs or singleton STs) where *n* is the total number of isolates for that serovar. *H* values above 0.0 indicate multiple eBGs/STs per serovar. Each dot corresponds to one or more serovars from Table S1 from which at least two isolates had been MLST typed. The sizes of the dots indicate the number of serovars for each data point with overlapping numbers of isolates and *H* values (see legend). Note that the abscissa is logarithmic rather than linear.
(PDF)

Figure S3 Radial dendrogram of 163 clusters of STs and 189 singleton STs found by ClonalFrame among concatenated sequences of seven housekeeping genes from 1,092 STs of *S. enterica* subspecies *enterica*. Each line represents a distinct ST, and groups of related STs are seen at the periphery of the dendrogram.
(PDF)

Figure S4 UPGMA tree of diversity within a 448 amino acid fragment of the FliC protein.
(PDF)

Figure S5 Variant nucleotides in a 1,320 bp fragment of the *fljB* gene. Position refers to the nucleotide position within the trimmed fragment, which starts 108 bp from the beginning of the intact gene in strain LT-2.
(PDF)

Figure S6 UPGMA tree of nucleotide diversity within a 1,320 bp fragment of the *fljB* gene.
(PDF)

Figure S7 UPGMA tree of diversity within a 440 amino acid fragment of the FljB protein.
(PDF)

Figure S8 Diversity versus frequency of S. *enterica* subspecies *enterica* isolates in France, the EU and the USA. Frequencies of serovars in pooled data over several years are plotted semi-logarithmically against *H* for each serovar as in Fig. S2. For parts B-D, all serovars are included and the numbers of discrete serovars at each position is indicated by different sized circles (see legend). Part A is based on the 29 most common serovars, none of which overlapped within the scattergram. Data were obtained from http://www.ecdc.europa.eu/en/activities/surveillance/TESSy/Pages/TESSy.aspx (A), internal records at the French National Reference Center for Salmonella, Institut Pasteur (B), as well as http://www.cdc.gov/ncidod/dbmd/phlisdata/salmonella.htm (C, D).
(PDF)

Table S1 eBurstGroups and singleton STs per serovar among 4,257 isolates of *S. enterica* subspecies *enterica*.
(XLSX)

Table S2 Serovars in 137 eBurstGroups containing 3,550 isolates of S. *enterica* subspecies *enterica*.
(XLSX)

Table S3 Antigenic formulas, eBGs and STs of serovars associated with Typhimurium.
(DOC)

Table S4 Antigenic formulas, eBGs and STs of serovars associated with Enteritidis and Dublin.
(DOC)

Table S5 Antigenic formulas, eBGs, STs and dTar status of serovars associated with Paratyphi B.
(DOC)

Table S6 Comparison of groupings from MLST versus MLEE and virulence tests for serovars Paratyphi B and var Java.
(XLSX)

Table S7 Properties of supposed 6,7:c:1,5 isolates.
(XLSX)

Table S8 Genbank accession codes and sequence groupings of *fliC* and *fljB* alleles.
(XLS)

Text S1 Deep phylogenetic structure and historical information regarding 6,7:c:1,5 isolates.
(DOCX)
