# Correction: Multilocus Sequence Typing as a Replacement for Serotyping in *Salmonella enterica*

**DOI:** 10.1371/journal.ppat.1009040

**Published:** 2020-10-21

**Authors:** Mark Achtman, John Wain, François-Xavier Weill, Satheesh Nair, Zhemin Zhou, Vartul Sangal, Mary G. Krauland, James L. Hale, Heather Harbottle, Alexandra Uesbeck, Gordon Dougan, Lee H. Harrison, Sylvain Brisse, the S. enterica MLST study group

Some URLs within the article are now inactive. The MLST website at University College Cork moved to University of Warwick in 2013 and was subsequently superseded by EnteroBase, which can be accessed at http://enterobase. warwick.ac.uk [2]. EnteroBase offers MLST genotyping on the basis of genomic short reads for all levels of MLST from 7-gene legacy MLST through core genome cgMLST to whole genome wgMLST. However, it no longer accepts new alleles based on ABI sequences as explained due to their excessive error rate. Allelic designations for sequences of 7-gene legacy MLST loci for *Salmonella enterica*, *Escherichia coli*, *Yersinia pseudotuberculosis* and *Moraxella catarrhalis* can be obtained from EnteroBase at http://enterobase.warwick.ac.uk/warwick_mlst_legacy.

The authors, however, now recommend using short read sequencing which is handled at http://enterobase.warwick.ac.uk [2].

Another URL in the original publication at PubMLST is now also no longer operative, and a general overview of MLST databases in general can be found at https://pubmlst.org/organisms.
