# Supplementary material for: Multilocus Sequence Typing as a Replacement for Serotyping in Salmonella enterica
Source: PLoS Pathog. 2012 Jun 21;8(6):e1002776. doi: 10.1371/journal.ppat.1002776 (PMC3380943; doi:10.1371/journal.ppat.1002776)
Supplement: Figure S1 — MSTree from Fig. 2 color-coded according to BAPS assignments to five clusters of allelic differences among 1097 STs. STs assigned to lineage 3 are colored in red and the four other colors indicate four other clusters of STs. Similar results were obtained with BAPS or STRUCTURE assignments to 5 clusters based on concatenated sequences of the seven MLST genes. The existence of STs from the other four clusters near the bottom of the figure is due to rare intermediate STs with recombinant alleles that artificially join lineage 3 to other clusters in a minimal spanning tree. (PDF) [file ppat.1002776.s001.pdf]

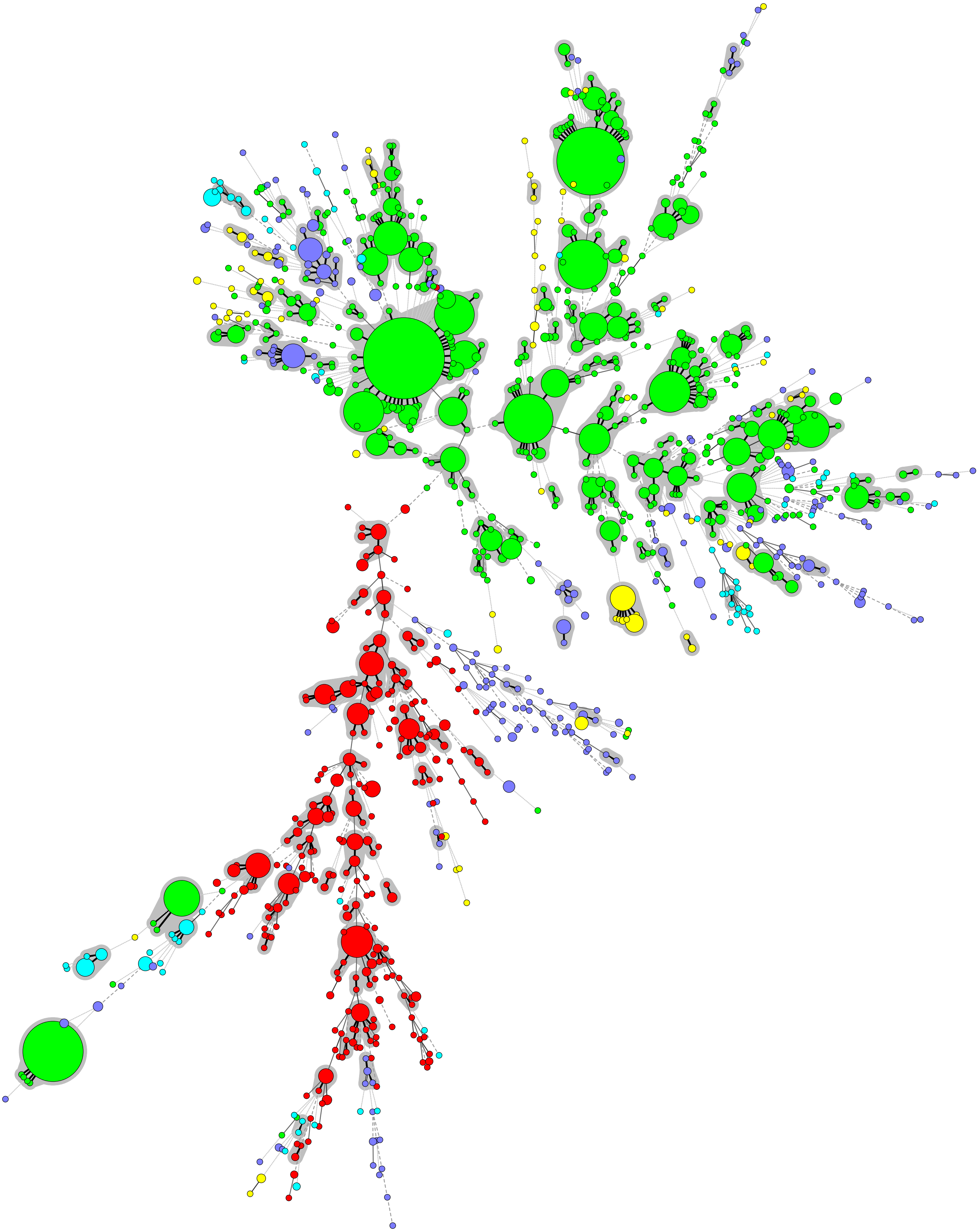

Supplementary Figure 1. MSTree from Fig. 2 color-coded according to BAPS assignments to five clusters of allelic differences among 1097 STs . STs assigned to lineage 3 are colored in red and the four other colors indicate the four other clusters of STs. Similar results were obtained with BAPS or STRUCTURE assignments to 5 clusters based on concatenated sequences of the seven MLST genes. The existence of STs from the other four groups near the bottom of the figure is due to rare intermediate STs with recombinant alleles that artificially join lineage 3 to other clusters in a minimal spanning tree.
