# Supplementary material for: Multilocus Sequence Typing as a Replacement for Serotyping in Salmonella enterica
Source: PLoS Pathog. 2012 Jun 21;8(6):e1002776. doi: 10.1371/journal.ppat.1002776 (PMC3380943; doi:10.1371/journal.ppat.1002776)
Supplement: Figure S2 — H, the index of genetic diversity, versus number of isolates per serovar in the MLST database. H was calculated as (n/(n-1))*(1.0 - the sum of squares of the relative frequency per serovar of isolates in discrete eBGs or singleton STs) where n is the total number of isolates for that serovar. H values above 0.0 indicate multiple eBGs/STs per serovar. Each dot corresponds to one or more serovars from Table S1 from which at least two isolates had been MLST typed. The sizes of the dots indicate the number of serovars for each data point with overlapping numbers of isolates and H values (see legend). Note that the abscissa is logarithmic rather than linear. (PDF) [file ppat.1002776.s002.pdf]

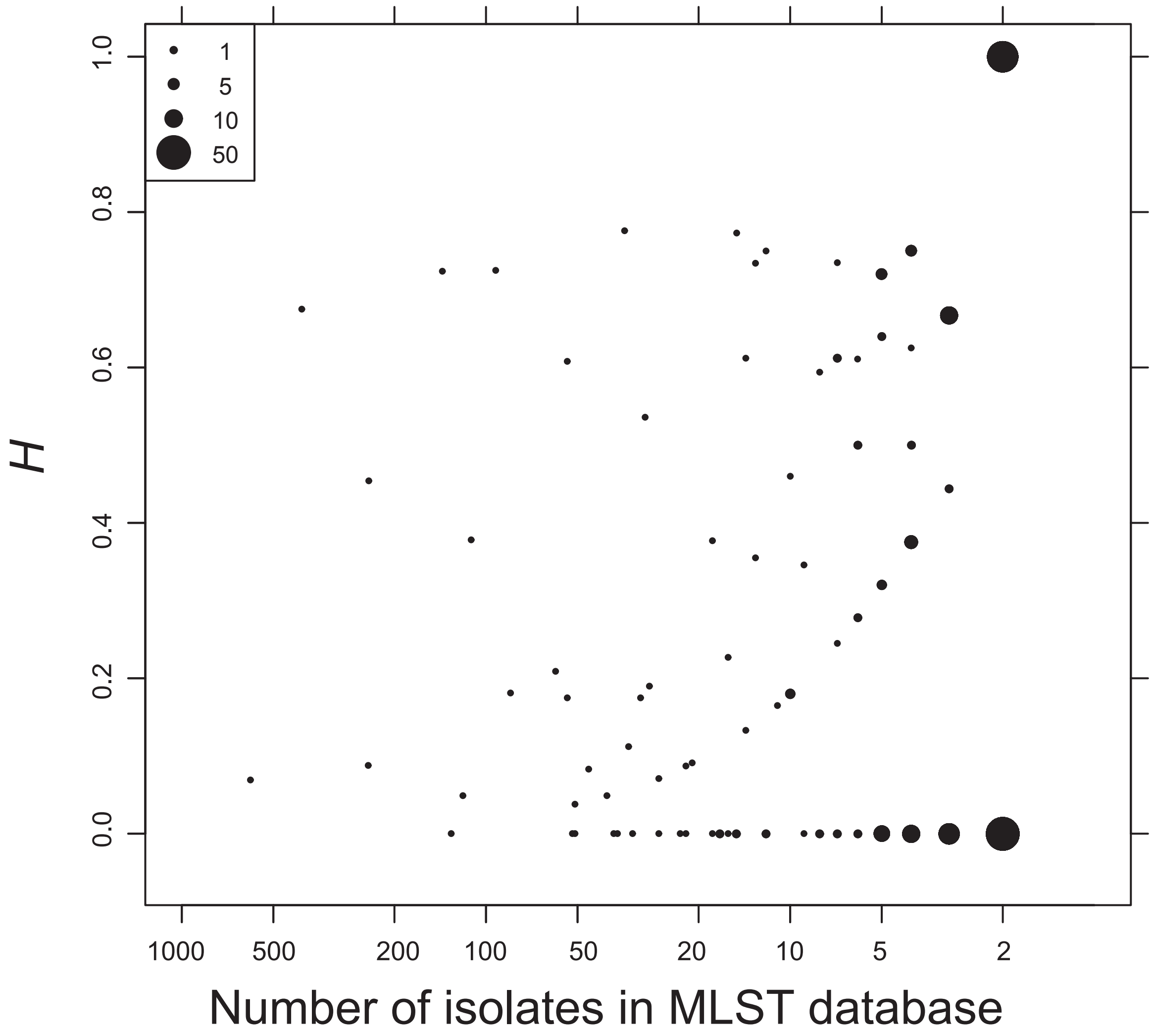

Supplementary Figure 2. *H*, the index of genetic diversity, versus number of isolates per serovar in the MLST database. *H* was calculated as (*n*/(*n*-1))*(1.0 - the sum of squares of the relative frequency per serovar of isolates in discrete eBGs or singleton STs) where *n* is the total number of isolates for that serovar. *H* values above 0.0 indicate multiple eBGs/STs per serovar. Each dot corresponds to one or more serovars from Table S1 from which at least two isolates had been MLST typed. The sizes of the dots indicate the number of serovars for each data point with overlapping numbers of isolates and *H* values (see legend). Note that the abscissa is logarithmic rather than linear.
