# Supplementary material for: Multilocus Sequence Typing as a Replacement for Serotyping in Salmonella enterica
Source: PLoS Pathog. 2012 Jun 21;8(6):e1002776. doi: 10.1371/journal.ppat.1002776 (PMC3380943; doi:10.1371/journal.ppat.1002776)
Supplement: Figure S3 — Radial dendrogram of 163 clusters of STs and 189 singleton STs found by ClonalFrame among concatenated sequences of seven housekeeping genes from 1,092 STs of S. enterica subspecies enterica. Each line represents a distinct ST, and groups of related STs are seen at the periphery of the dendrogram. (PDF) [file ppat.1002776.s003.pdf]

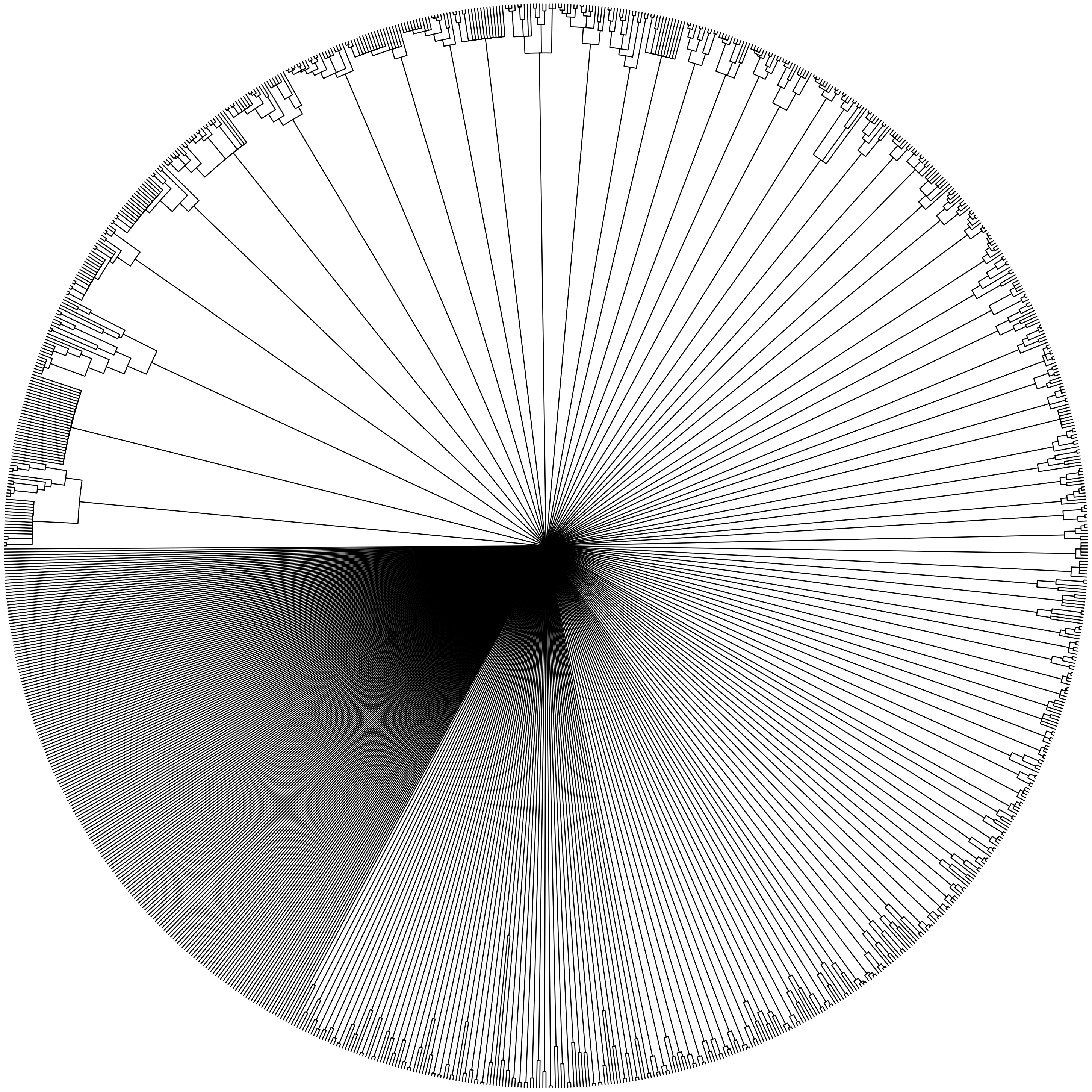

Supplementary Figure 3. Radial dendrogram of 163 clusters of STs and 189 singleton STs found by ClonalFrame among concatenated sequences of seven housekeeping genes from 1092 STs of *S. enterica* subspecies *enterica*. Each line represents a distinct ST, and groups of related STs are seen at the periphery of the dendrogram.
