# Supplementary material for: Multilocus Sequence Typing as a Replacement for Serotyping in Salmonella enterica
Source: PLoS Pathog. 2012 Jun 21;8(6):e1002776. doi: 10.1371/journal.ppat.1002776 (PMC3380943; doi:10.1371/journal.ppat.1002776)
Supplement: Figure S4 — UPGMA tree of diversity within a 448 amino acid fragment of the FliC protein. (PDF) [file ppat.1002776.s004.pdf]

FliC Amino Acid UPGMA Tree

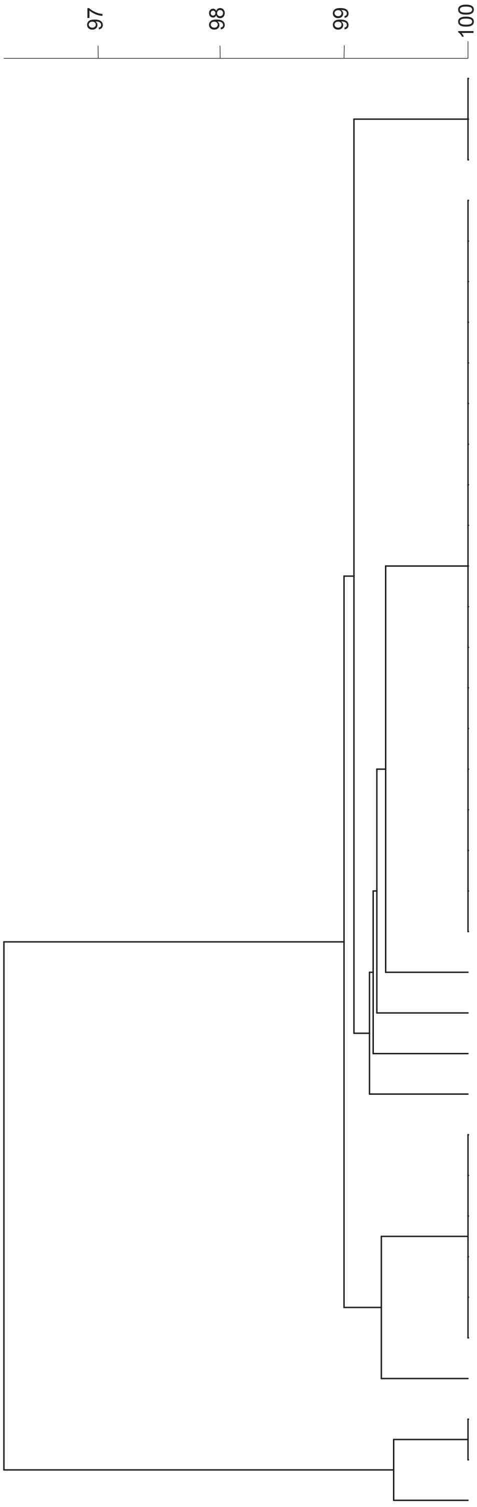

Strain	Serotype	MLST ST	MLST eBG	FliC group
6631/88	Choleraesuis s.s.	68	6	
34K	Choleraesuis s.s.	139	6	C
CDC34	Choleraesuis			
CDC35	Choleraesuis			
SARB4	Choleraesuis Kunzendorf	66	6	
9520/04	Choleraesuis Kunzendorf	66	6	
48-0391	Choleraesuis Kunzendorf	133	6	
5176/85	Choleraesuis Kunzendorf	145	6	
37K	Choleraesuis Kunzendorf	246	6	
RKS1263	Choleraesuis Kunzendorf	363	6	
211K	Choleraesuis Kunzendorf	497	6	
06-5620	Choleraesuis Kunzendorf	630	6	
VCMM3420	Paratyphi C			
9519/04	Choleraesuis Kunzendorf	634	6	
SARB48	Paratyphi C	90	20	A
SARB49	Paratyphi C	114	20	
7972/93	Paratyphi C	146	20	
8723/97	Typhisuis	147	20DLV2	
38K	Typhisuis	636	20DLV	
9761/07	Decatur	637		
SARB7	Decatur	69	142	
6563/88	Decatur	635	142	
AF336929	Bury			
CH12440	Choleraesuis			
CDC621	Jericho			
1435K	Paratyphi C	90	20	
SARB5	Decatur	67	141	
2/84	Decatur	631	141	
98-02313	Decatur	186	144	
06-3890	Decatur	632	144	B
1/74	Decatur	633		
CDC696	Goeteborg			
SARB8	Decatur	70		
CDCAr468	IIIb 57:c:z:z60			
CDCAr429	IIIb 41:c:e,n,x,z15			D
CDCAr548	IIIb 57:c:e,n,x,z15			

Supplementary Figure 4. UPGMA tree of diversity within a 448 amino acid fragment of the FliC protein.
