# Supplementary material for: Multilocus Sequence Typing as a Replacement for Serotyping in Salmonella enterica
Source: PLoS Pathog. 2012 Jun 21;8(6):e1002776. doi: 10.1371/journal.ppat.1002776 (PMC3380943; doi:10.1371/journal.ppat.1002776)
Supplement: Figure S5 — Variant nucleotides in a 1,320 bp fragment of the fljB gene. Position refers to the nucleotide position within the trimmed fragment, which starts 108 bp from the beginning of the intact gene in strain LT-2. (PDF) [file ppat.1002776.s005.pdf]

fljB variant nucleotides

Strain	Serotype	MLST ST	MLST eBG	Position	FljB Group
				.... 111111111 1111111111 1111111111 1111111111 1111111111 111111111	
				.... 111111 1122222222 3333333333 3333444444 4444445555 5555555555 5555555555 5555666666 6666666666 6667777777 7777777777 7777888888 8888888889 9999999999 9000000000 0111111111 1111111111 1111222222 2222222222 222222333	
				122355667 7899011223 6700122447 0012234556 6889005566 6799990001 1122223334 4444555556 6689112333 3356678899 9990111222 2335556667 7778011122 2333348890 2224567778 9022456679 9012233455 6677788888 9999001112 2334455666 677779011	
				8217947362 4409814032 5417658096 3971798570 3679583808 9912582380 3605781891 3467015892 5731270123 4580371401 3465479056 7255785670 3789136703 8378905816 1470161783 0539405848 9795805004 6701625689 1246010254 9072314347 801256248	
SL3261	Typhimurium			ACCGGCCCCT TTTCCCTGCC TACCCCCCCC CGACCCCATC TCTTCAAAGC ACTAGTGAGA TTGTCGTAAG TAGGTCACGG GGGCTTCGCC GCACTCACGG TGCGGTCTCG ATAAGAGGCT TAGATTCGAG TGTGAGACTT CCAGACGCTC CGCCCTCCCC AAGACCACCA CCGCGGTGGC GCCCTCGAAC CTACCTCTCT CGAAGCCTA	
CDC1982	Derby			....A......	
CDC000477	Stanley			.....T.... ....T..T.TT. .....T.... ....A...... ....C..	
CDC000377	Muenchen			.....T.... ....T..T.TT. .....T.... ....A...... ....C..	
CDC000462	Litchfield			.....T.... ....T..T.TT. .....T.... ....A...... ....C..	
CDC010148	4,12:1,2:1,2			....A......	
SPB7	Paratyphi B			....A....C ....T..T.TT. .....T.... ....G... ....A...... .... .A........ .... .C.....C..	
SL254	Newport			.....T.... ....T..T.TT. .....T.... ....A...... ....C..	
SL476	Heidelberg			.....T.... ....T..T.TT. .....T.... ....A...... ....C..	
84	Saintpaul			.....T.... ....T.... ....A...... ....C..	A (1,2)
FSLS9-102	uncertain			....A......	
CDC02296	(1),4,5,12:i:1,2,7			.... .T........ ....A......	
1388_04	Paratyphi B var. Java			....A....C ....T..T.TT. .....T.... ....G... ....A...... .... .A........ .... .C.....C.. T........	
N1343_04	Paratyphi B			....A....C ....T..T.TT. .....T.... ....G... ....A...... .... .A........ .... .C.....C.. T........	
3474_02	Paratyphi B var. Java			.....T.... .....T.... ....T. .....T.... ....A...... .... T......C..	
CDC16	Heidelberg			....C....C.. ....A......	
SARB7	Decatur	69	142	.T...T.... ....T.... .... G.....AC.. ..A.....C. ....G..... ....A...... ....C..	
6563/88	Decatur	635	142	.T...T.... ....T.... .... G.....AC.. ..A.....C. ....G..... ....A...... ....C..	B (1,5)
9520/04	Choleraesuis Kunzendorf	66	6	.....T.... ....TT.... ....TT ...T.T.... ....A. .C..TA..C. ....G....A ....A...... .... .A........ .... .C.....C..	
48-0391	Choleraesuis Kunzendorf	133	6	.....T.... ....TT.... ..TA.T..TT .....T.... ....A. .C..TA..C. ....G....A ....A...... .... .A........ .... .C.....C..	
5176/85	Choleraesuis Kunzendorf	145	6	.....T.... ....TT.... ..TA.T..TT .....T.... ....A. .C..TA..C. ....G....A ....A...... .... .A........ .... .C.....C..	
37K	Choleraesuis Kunzendorf	246	6	.....T.... ....TT.... ..TA.T..TT .....T.... ....A. .C..TA..C. ....G....A ....A...... .... .A........ .... .C.....C..	
RKS1263	Choleraesuis Kunzendorf	363	6	.....T.... ....TT.... ..TA.T..TT .....T.... ....A. .C..TA..C. ....G....A ....A...... .... .A........ .... .C.....C..	
06-5620	Choleraesuis Kunzendorf	630	6	.....T.... ....TT.... ..TA.T..TT .....T.... ....A. .C..TA..C. ....G....A ....A...... .... .A........ .... .C.....C..	
9519/04	Choleraesuis Kunzendorf	634	6	.....T.... ....TT.... ....TT ...T.T.... ....A. .C..TA..C. ....G....A ....A...... .... .A........ .... .C.....C..	
6631/88	Choleraesuis s. s.	68	6	.....T.... ....TT.... ..TA.T..TT .....T.... ....A. .C..TA..C. ....G....A ....A...... .... .A........ .... .C.....C..	
34K	Choleraesuis s. s.	139	6	.....T.... ....TT.... ..TA.T..TT .....T.... ....A. .C..TA..C. ....G....A ....A...... .... .A........ .... .C.....C..	
SARB48	Paratyphi C	90	20	.....T.... ....TT.... ..TA.T..TT .....T.... ....A. .C..TA..C. ....G....A ....A...... .... .A........ .... .C.....C..	
SARB49	Paratyphi C	114	20	.....T.... ....TT.... ..TA.T..TT .....T.... ....A. .C..TA..C. ....G....A ....A...... .... .A........ .... .C.....C..	C (1,5)
7972/93	Paratyphi C	146	20	.....T.... ....TT.... ..TA.T..TT .....T.... ....A. .C..TA..C. ....G....A ....A...... .... .A........ .... .C.....C..	
38K	Typhisuis	636	20DLV	.....T.... ....TT.... ..TA.T..TT .....T.... ....A. .C..TA..C. ....G....A ....A...... .... .A........ .... .C.....C..	
8723/97	Typhisuis	147	20DLV2	.....T.... ....TT.... ..TA.T..TT .....T.... ....A. .C..TA..C. ....G....A ....A...... .... .A........ .... .C.....C..	
B20	Choleraesuis			.....T.... .C..TT.... ....TT ...T.T.... ....A. .C..TA..C. ....G....A ....A...... .... .A........ .... .C.....C..	
SC-B67	Choleraesuis Kunzendorf	66	6	.....T.... ....TT.... ..TA.T..TT .....T.... ....A. .C..TA..C. ....G....A ....A...... .... .A........ .... .C.....C..	
1435K	Paratyphi C	90	20	.....T.... ....TT.... ..TA.T..TT .....T.... ....A. .C..TA..C. ....G....A ....A...... .... .A........ .... .C.....C..	
N1258_99	Paratyphi C			.....T.... ....TT.... ..TA.T..TT .....T.... ....A. .C..TA..C. ....G....A ....A...... .... .A........ .... .C.....C.. T........	
ATCC7001	Choleraesuis			.....T.... ....TT.... ..TA.T..TT .....T.... ....A. .C..TA..C. ....C....A ....A...... ....C... .... .A........ .... .C.....C..	
CH12440	Choleraesuis			.....T.... C...TT.... ....TT ...T.T.... .....GG... ....A. .C..TA.... ....G....A ..A....... ....A...... .... .A........ ......G... .... .C.....C.. T........	
CDC2532	43:e,h:1,5			.....T.... ...TTT.AT. .....T.... ...T.T.... ..G.T...A. GTCCAC.... CC.CTT..C. ....G.TG.. AA..G.G... ....C..... ...A...... .C........ ....T.C .... .GT....... .... .C.....C.. ....C.	D (1,5)
CDC000499	Thompson			....T..... G.CCAC...G CC.CTT..C. ....G..G.. AA.T...... .....ATAT. G.TA...... G.....A... ...G...... ....C..	
CDC1498	Beaudesert			.....T.... ....T....T .A...T.... ..G.T..... .... .GACAG.A.A ....CT. .A........ ...A...... ......A... ....C..AG. .... .T........ ...T...... ..T....... ....C...G. .C.....C..	
ATCC10727	Florida			.... .C........ ....T.... ..G.T..... .... .GACAG.A.A ....CT. .A........ ...A...... ......A... ....C..AG. .... .T........ ...T...... ..T....... ....C..	
CDC2473	Pomona			.....T.... ....G...... .....T.... ..G.T..... .... .GACAG.A.A ....CT. .A........ ...A...... ......A... ....C..AG. ....T.. ....T.. ..T....... ....T.C...GT .C.....C.. .....TT..	
CDC112	Bredeney			.....T.... .C........ ....TT... ....T..... .... .GACAG.A.A ....CT. .A........ ...A...... .... .C........ ....C. ....T.T .GT....... ....T.C...GT .C.....C.. .....TT..	
CDC2206	Nola			........T. ....TT.ATT ....T.... ....T..... .... .GACAG.A.A ....CT. .A........ ...A...... ....C. ....T.T .GT....... .....T.... ....C...G. .C.....C.. ....C.	E (1,7)
CDC2518	Give			.....T.... .C........ ....TT... ....T..... .... .GACAG.A.A ....CT. .A........ ...A...... .... .C........ ....C. ....T.T .GT....... ....T.C...GT .C.....C.. .....TT..	
CVM19633	Schwarzengrund			.....T.... .C........ ....TT... ....T..... .... .GACAG.A.A ....CT. .A........ ...A...... .... .C........ ....C. ....T.T .GT....... ....T.C...GT .C.....C.. .....TT..	
38_05	Indiana			.....T..T. ....TA....T. G....T.... .... .GACAG.A.A ....CTT .A........ ...A...... ....T....... .... A..T...... T......C.. ......T..	
6163_04	Indiana			.....T..T. ....TA....T. G....T.... .... .GACAG.A.A ....CTT .A........ ...A...... ....T....... .... A..T...... T......C.. T.....T..	
1370_05	Indiana			C....T..T. ..A....... ..TA....T. G....T.... .... .GACAG.A.A ....CTT .A........ ...A...... ....T....... ....C .... A......... ..T....C.. ......T..	
CDC1863	Kambole			.....T.... ....TT.AT. ..T....... .....TT... ....T..... .... .GACAG.T.A ....ATATA G.GA...... G.....A... ..AGCG...A ..C....... .T........ ....T....... .... .C.....C.. ......T..	F (1,2,7)
CDC1967	Eingedi			.....T.... ....TT.AT. ..T....... .....TT... ....T..... .... .GACAG.T.A ....ATATA G.TA...... G...T.A... ..AGCG...A ..C....... .T........ ....T....... .... .C.....C.. ......T..	
ST163	Coeln			....A..T... ....T. C........C ....T....... T.......C	G (1,2)
CDC513	Agama			.....T.... ....T..T.TT. .....T.... ....T.C.A. G.CCAC..A. .C..TA.CT. C......... AA.T.A.... A...CA...C ...AAG.A.T .C.....A.. ......T... ....T...T.. ..T....... .... .C.....C.. ......TC.	
CDC1994	Poona			.....T.... ....G...... .....T.... ....T...A. G.CCAC..A. .C..TA.CT. C......... AA.T.A.... A...CA...C ...AAG.A.T .C.....A.. ......T... ....C.... ..T....... ...T...... ....T .C.....C..	
150_05	Agama			.....T.... .... G.T..T.TT. .....T.... ....T.CGA. G.CCAC..A. .C..TA.CT. C......... AA.T.A.... A...CA...C ...AAG.A.T .C.....A.. ......T... ....T...T.. ..T......C ....T.... .CT....C.. T.....TCC	H (1,6)
ATCC9270	Anatum			.....T.... ....T..T.TT. .....T.... ....T.C.A. G.CCAC..A. .C..TA.CTA C......... AA.T.A.... A...CA...C ...AAG.A.T .C.....A.. ......T... ......C... ....T...... ..T..A..A. A......... .AA.C.A.G. ....C..	
CDC1186	II 42:e,n,x:1,6			..T...TT.. ....TT.... ....T..... C..CT..... G.CCAC..A. .C..TA.CT. ....A.. .....A.... A....A.A.. ...A...... ..G....... .T..C..A.. ....CA ..GAT....A T...T..... .GT....... .TTTATCA.T A.T.CT.... .C...C.C.A .TCTC..C.	
CDCAr555	IIIb 40:z39:1,6			.........C ....TT.... .....TT... .....T..C. ....T....A G.CCAC..A. .C..TA.CT. ....A.. .....A.... A..T.A.A.C ...A...... ..G....... .T..C..A.. ....ACA T.GA.....A T......... .GT....... ....T ..T.C...G. .C...TCT.. ....C.	I (1,6)
CDC380	II 17:e,n,x,z15:1,6			...A.....C ....TT.... .G......T. ....T ....T..... G.CCAC..A. .C..TA.CT. ....A.. .....A.... A.G....A.. ...A...... ..G.AC..TC C...C..AG. ....ACC ..G......A T......... .G.GT..T.. ....A. ..T.C..TG. .C.T...CT. ....C.	
ATCC9150	Paratyphi A			.....T.... ......C... ..T......T .....T.... ...CT..... G.CCAC...G CC.CTT..C. ....G..GA. AA.T...... .....ATATA GATA...... G.....A... ...GC..AG. ACGACA.... .T...T.... .... .C..T..C..	
N2761_03	Paratyphi A			.....T.... ......C... ..T......T .....T.... ...CT..... G.CCAC...G CC.CTT..C. ....G..GA. AA.T...... .....ATATA GATA...... G.....A... ...GC..AG. ACGACA.... .T...T.... .... .C..T..C.. T........	
N1996_04	Paratyphi A			.....T.... ......C... ..T......T .....T.... .T.CT..... G.CCAC...G CC.CTT..C. ....G..GA. AA.T...... .....ATATA GATA...... G.....A... ...GC..AG. ACGACA.... .T...T.... .... .C..T..C.. T........	J (1,5)
1/74	Decatur	633		....T..... G.CCAC...G CC.CTT..C. ....G..G.. AA.T...... .....ATATA G.TA...... G.....A... ...GC..AG. .CGACA.... .T........ ...T..A... ..T....... ....T.C...G. ....C.. ....C.	
CDC2201	Bovismorbificans			....TT.AT. ....T .....T.... ...CT..C.. G.....AC.. ..A.....C. ....G.G... ....A....TA ...CAC..T. C...C..AG. .C.ACA.... ....T...T.T .GT....... ....T.C...GT .C.....C.. .....TT..	
9761/07	Decatur	637		....T. .....T.... ....T..C.. G.....AC.. ..A.....C. ....G.G... ....A....TA ...CAC..T. C...C..AG. .C.ACA.... ....T...T.T .GT....... ....T.C...GT .C.....C.. .....TT..	
CDC020017	Rough:r:1,5			.....T.... ....TT.... ....T.... ....T..C.. G.....AC.. ..A.....C. ....A....T. ...CAC..T. C..GC..AG. .CGACA.... ....T...... ..T....... .A.T...... ....C...G. .C.....C.. ......TC.	
CDC1428	Infantis			.....T.... ....TT.... ....T.... ....T..C.. G.....AC.. ..A.....C. ....A....T. ...CAC..T. C..GC..AG. .CGACA.... ....T...... ..T....... .A.T...... ....C...G. .C.....C.. ......TC.	
SARB8	Decatur	70		.....T.... ....TT.AT. ....T.... ....T..C.. G.....AC.. ..A.....C. ....A....T. ...CAC..T. C..GC..AG. .CGACA.... ....T...... ..T....... ....C...G. .C.....C.. ......TC.	K (1,5)
SARB5	Decatur	67	141	.....T.... ....T..T..T. .....T.... ....T..C.. G.....AC.. ..A.....C. ....A....T. ...CAC..T. C..GC..AG. .CGACA.... ......C... ...T...... ..T....... ....C...G. .C.....C.. ......TC.	
2/84	Decatur	631	141	.....T.... ....T..T..T. .....T.... ....T..C.. G.....AC.. ..A.....C. ....A....T. ...CAC..T. C..GC..AG. .CGACA.... ......C... ...T...... ..T....... ....C...G. .C.....C.. ......TC.	
98-02313	Decatur	186	144	.....T.... ....T..T..T. .....T.... ....T..C.. G.....AC.. ..A.....C. ....A....T. ...CAC..T. C..GC..AG. .CGACA.... ......C... ...T...... ..T....... ....C...G. .C.....C.. ......TC.	
06-3890	Decatur	632	144	.....T.... ....T..T..T. .....T.... ....T..C.. G.....AC.. ..A.....C. ....A....T. ...CAC..T. C..GC..AG. .CGACA.... ......C... ...T...... ..T....... ....C...G. .C.....C.. ......TC.	

Supplementary Figure 5. Variant nucleotides in a 1320 bp fragment of the *fljB* gene. Position refers to the nucleotide position within the trimmed fragment, which starts 108 bp from the beginning of the intact gene in strain LT-2.
