# Supplementary material for: Multilocus Sequence Typing as a Replacement for Serotyping in Salmonella enterica
Source: PLoS Pathog. 2012 Jun 21;8(6):e1002776. doi: 10.1371/journal.ppat.1002776 (PMC3380943; doi:10.1371/journal.ppat.1002776)
Supplement: Figure S6 — UPGMA tree of nucleotide diversity within a 1,320 bp fragment of the fljB gene. (PDF) [file ppat.1002776.s006.pdf]

fljB Nucleotide UPGMA Tree

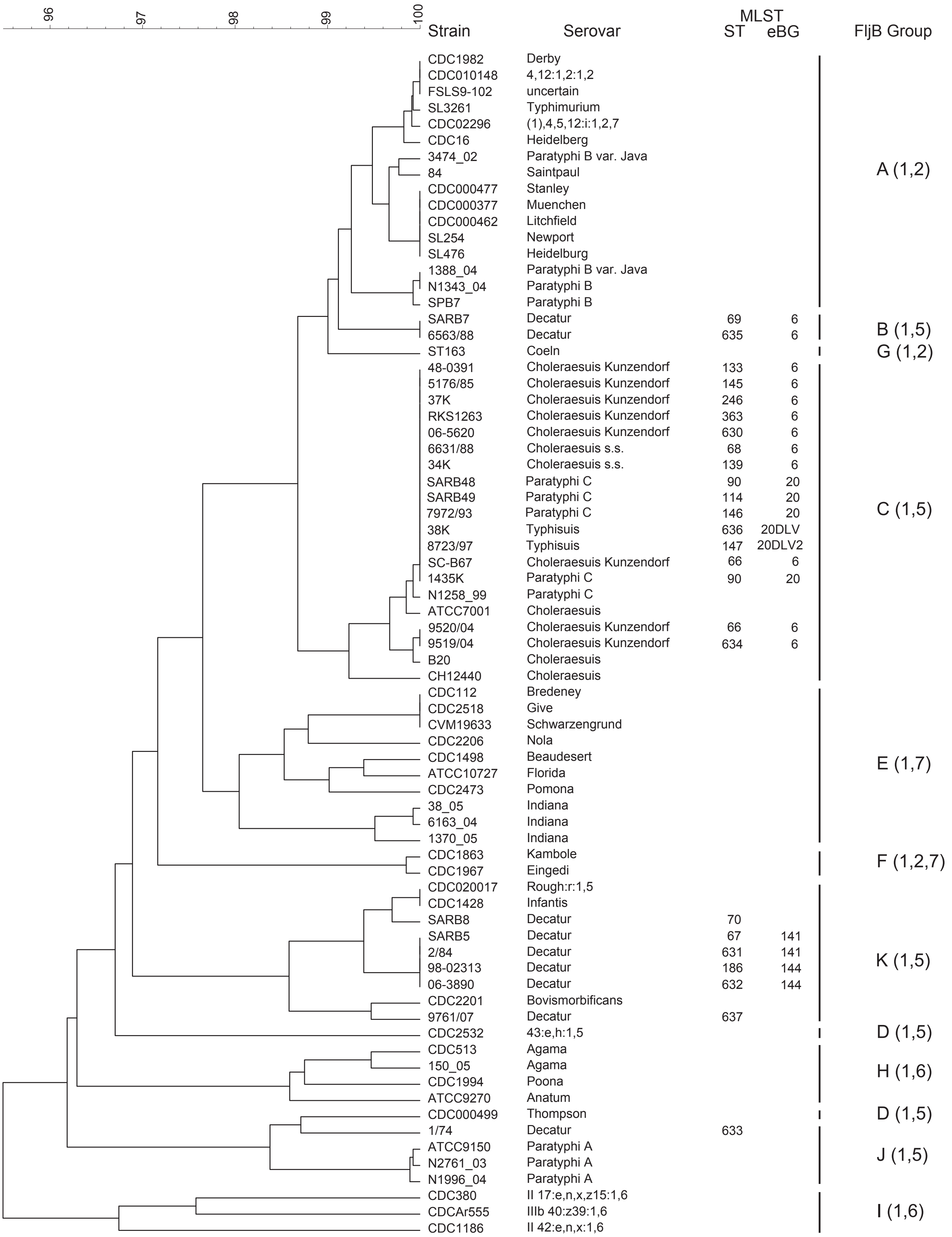

Supplementary Figure 6. UPGMA tree of nucleotide diversity within a 1320 bp fragment of the *fljB* gene.
