# Supplementary material for: Multilocus Sequence Typing as a Replacement for Serotyping in Salmonella enterica
Source: PLoS Pathog. 2012 Jun 21;8(6):e1002776. doi: 10.1371/journal.ppat.1002776 (PMC3380943; doi:10.1371/journal.ppat.1002776)
Supplement: Figure S7 — UPGMA tree of diversity within a 440 amino acid fragment of the FljB protein. (PDF) [file ppat.1002776.s007.pdf]

FljB amino acid UPGMA tree

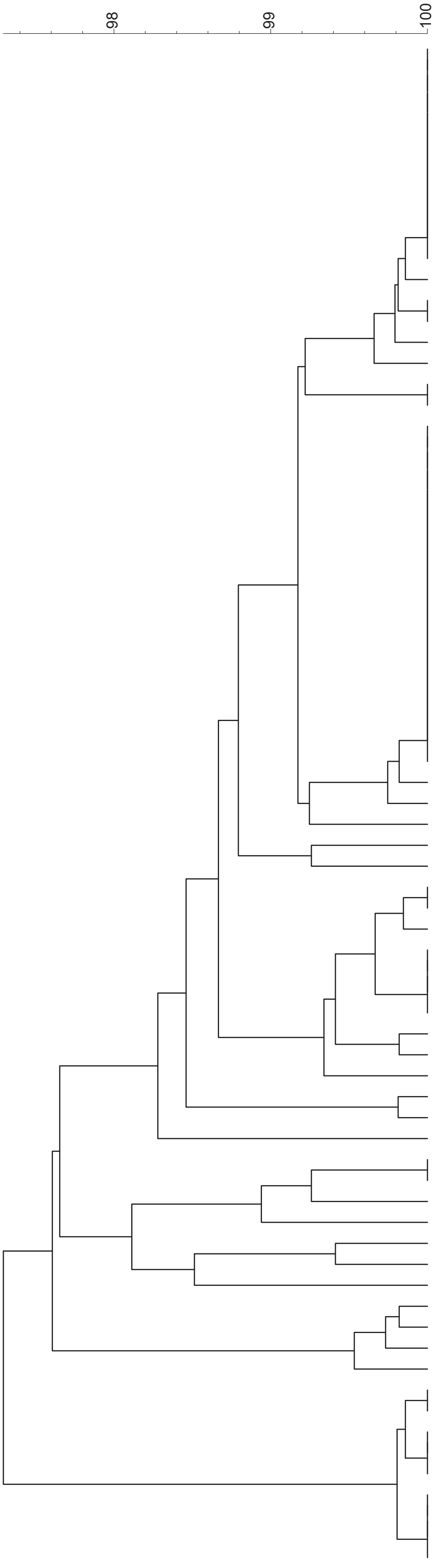

Strain	Serotype	MLST ST	MLST eBG	FljB Group (phase II antigens)
SL3261	Typhimurium			A (1,2)
CDC1982	Derby			
CDC000477	Stanley			
CDC000377	Muenchen			
CDC000462	Litchfield			
CDC010148	4,12:1,2:1,2			
SPB7	Paratyphi B			
SL254	Newport			
SL476	Heidelburg			
84	Saintpaul			
FSLS9-102	uncertain			
CDC02296	(1),4,5,12:i:1,2,7			
1388_04	Paratyphi B var. Java			
N1343_04	Paratyphi B			
3474_02	Paratyphi B var. Java			
CDC16	Heidelberg			
SARB7	Decatur	69	142	B (1,5)
6563/88	Decatur	635	142	
9520/04	Choleraesuis Kunzendorf	66	6	C (1,5)
48-0391	Choleraesuis Kunzendorf	133	6	
5176/85	Choleraesuis Kunzendorf	145	6	
37K	Choleraesuis Kunzendorf	246	6	
RKS1263	Choleraesuis Kunzendorf	363	6	
06-5620	Choleraesuis Kunzendorf	630	6	
9519/04	Choleraesuis Kunzendorf	634	6	
6631/88	Choleraesuis s.s.	68	6	
34K	Choleraesuis s.s.	139	6	
SARB48	Paratyphi C	90	20	
SARB49	Paratyphi C	114	20	
7972/93	Paratyphi C	146	20	
38K	Typhisuis	636	20DLV	
8723/97	Typhisuis	147	20DLV2	
B20	Choleraesuis			
SC-B67	Choleraesuis Kunzendorf	66	6	
1435K	Paratyphi C	90	20	
N1258_99	Paratyphi C			
ATCC7001	Choleraesuis			
CH12440	Choleraesuis			
CDC2532	43:e,h:1,5			D (1,5)
CDC000499	Thompson			
CDC1498	Beaudesert			E (1,7)
ATCC10727	Florida			
CDC2473	Pomona			
CDC112	Bredeney			
CDC2206	Nola			
CDC2518	Give			
CVM19633	Schwarzengrund			
38_05	Indiana			
6163_04	Indiana			
1370_05	Indiana			
CDC1863	Kambole			F (1,2,7)
CDC1967	Eingedi			
ST163	Coeln			G (1,2)
CDC513	Agama			H (1,6)
CDC1994	Poona			
150_05	Agama			
ATCC9270	Anatum			
CDC1186	II 42:e,n,x:1,6			I (1,6)
CDCAr555	IIIb 40:z39:1,6			
CDC380	II 17:e,n,x,z15:1,6			
ATCC9150	Paratyphi A			J (1,5)
N2761_03	Paratyphi A			
N1996_04	Paratyphi A			
1/74	Decatur	633		
CDC2201	Bovismorbificans			K (1,5)
9761/07	Decatur	637		
CDC020017	Rough:r:1,5			
CDC1428	Infantis			
SARB8	Decatur	70		
SARB5	Decatur	67	141	
2/84	Decatur	631	141	
98-02313	Decatur	186	144	
06-3890	Decatur	632	144	

Supplementary Figure 7. UPGMA tree of diversity within a 440 amino acid fragment of the FljB protein.
