# Supplementary material for: Multilocus Sequence Typing as a Replacement for Serotyping in Salmonella enterica
Source: PLoS Pathog. 2012 Jun 21;8(6):e1002776. doi: 10.1371/journal.ppat.1002776 (PMC3380943; doi:10.1371/journal.ppat.1002776)
Supplement: Figure S8 — Diversity versus frequency of S. enterica subspecies enterica isolates in France, the EU and the USA. Frequencies of serovars in pooled data over several years are plotted semi-logarithmically against H for each serovar as in Fig. S2. For parts B-D, all serovars are included and the numbers of discrete serovars at each position is indicated by different sized circles (see legend). Part A is based on the 29 most common serovars, none of which overlapped within the scattergram. Data were obtained from http://www.ecdc.europa.eu/en/activities/surveillance/TESSy/Pages/TESSy.aspx (A), internal records at the French National Reference Center for Salmonella, Institut Pasteur (B), as well as http://www.cdc.gov/ncidod/dbmd/phlisdata/salmonella.htm (C, D). (PDF) [file ppat.1002776.s008.pdf]

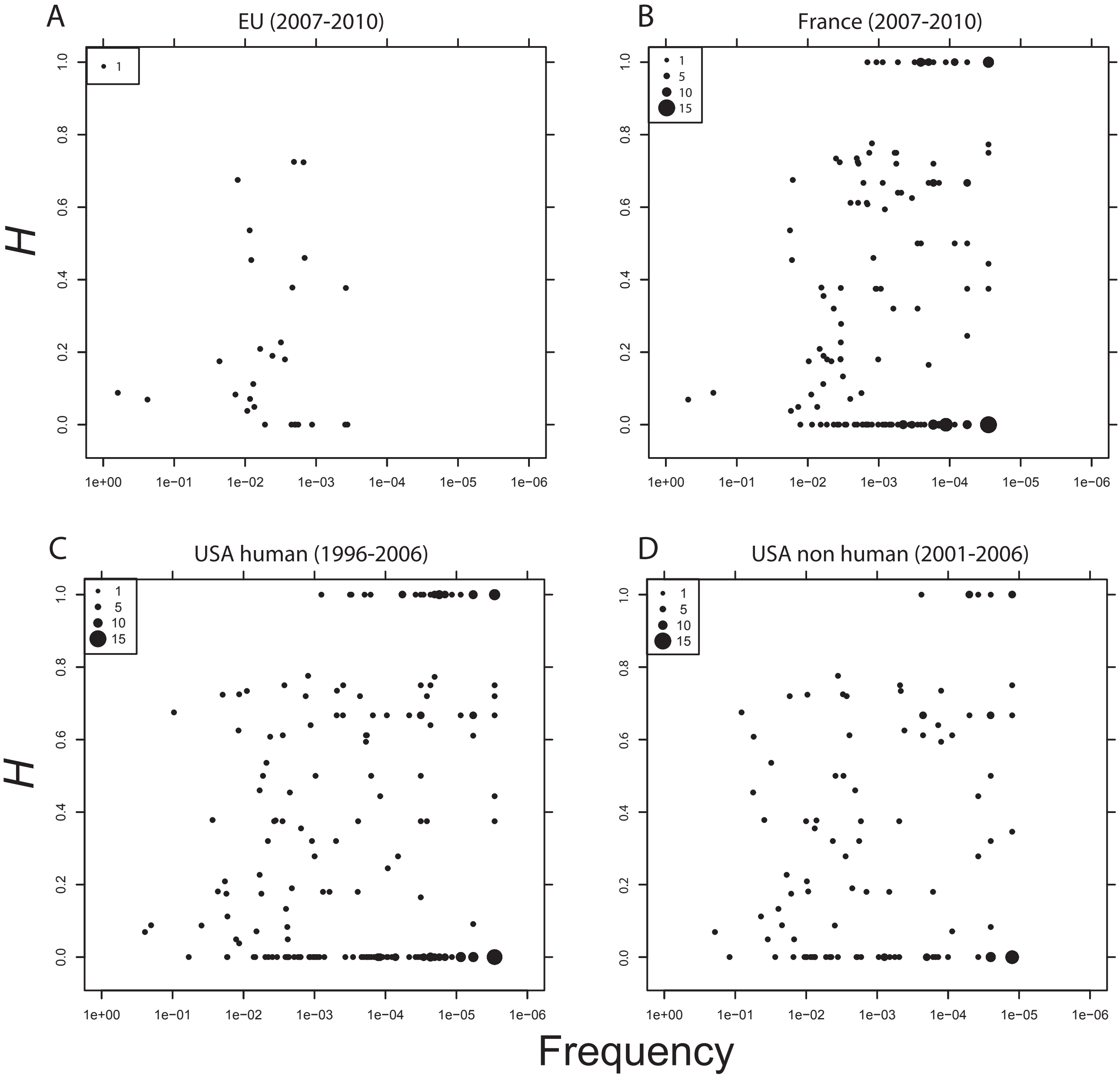

Supplementary Figure 8. Diversity versus frequency of *S. enterica* subspecies *enterica* isolates in France, the EU and the USA. Frequencies of serovars in pooled data over several years are plotted semi-logarithmically against *H* for each serovar as in Fig. S2. For parts B-D, all serovars are included and the numbers of discrete serovars at each position is indicated by different sized circles (see legend). Part A is based on the 29 most common serovars, none of which overlapped within the scattergram. Data were obtained from http://www.ecdc.europa.eu/en/activities/surveillance/TESSy/Pages/TESSy.aspx (A), internal records at the French National Reference Center for Salmonella, Institut Pasteur (B), as well as http://www.cdc.gov/ncidod/dbmd/phlisdata/salmonella.htm (C, D).
