# Supplementary material for: Multilocus Sequence Typing as a Replacement for Serotyping in Salmonella enterica
Source: PLoS Pathog. 2012 Jun 21;8(6):e1002776. doi: 10.1371/journal.ppat.1002776 (PMC3380943; doi:10.1371/journal.ppat.1002776)
Supplement: Table S3 — Antigenic formulas, eBGs and STs of serovars associated with Typhimurium. (DOC) [file ppat.1002776.s011.doc]

Supplementary Table 3. Antigenic formulas, eBGs and STs of serovars associated with Typhimurium

| Serovar | Antigenic formula | eBG | ST | Citation |
| --- | --- | --- | --- | --- |
| Typhimurium | [1],4,[5],12:i:1,2 | eBG1 eBG138 ST513 | ST19 plus 25 others | [38,39,99] |
| Monophasic Typhimurium | [1],4,[5],[12]:i:- | eBG1 eBG138 | ST19 ST34 | [54,85,100–104] |
| Monophasic Typhimurium | 4, [5],12:-:1,2 | eBG1 eBG138 | ST19 ST34 |  |
| Hato | [1],4,[5],12:g,m,s:[1,2] | eBG1 ST1056 ST1329 | ST19 ST1056 ST1329 | [105] |
| Farsta | 4,12:i:e,n,x | eBG1 ST886 | ST19 ST886 | [54] |
| Kunduchi | [1],4,[5],12,[27]:l,z28:1,2 |  | ST513 ST741 ST1014 ST1206 | [105] |
