# Supplementary material for: Multilocus Sequence Typing as a Replacement for Serotyping in Salmonella enterica
Source: PLoS Pathog. 2012 Jun 21;8(6):e1002776. doi: 10.1371/journal.ppat.1002776 (PMC3380943; doi:10.1371/journal.ppat.1002776)
Supplement: Table S4 — Antigenic formulas, eBGs and STs of serovars associated with Enteritidis and Dublin. (DOC) [file ppat.1002776.s012.doc]

Supplementary Table 4. Antigenic formulas, eBGs and STs of serovars associated with Enteritidis and Dublin

| Serovar | Antigenic formula | eBG | ST |
| --- | --- | --- | --- |
| Enteritidis | [1],9,12:[f],g,m,[s],[t]:[1,7] | eBG4, eBG93, eBG32, ST77, ST6 | ST11 plus 12 STs, ST180 plus 1 ST, ST74, ST77, ST6 |
| Enteritidis non-motile | [1],9,12:-:- | eBG4 | ST11, ST814 |
| Rosenberg | 9,12:g,z_85_:- | eBG4 | ST11 |
| Moscow | [1],9,12:g,q:- | eBG4 | ST11, ST1400 |
| Blegdam | 9,12,g,m,q:- | eBG4 | ST739 |
| Antarctica | 9,12:g,z_63_:- | eBG4 | ST11 |
| Nitra | 2,12:g,m,:- | eBG4 | ST11 |
| Gallinarum | [1],9,12:-:- | eBG4 | ST78, ST331, ST470, ST762 |
| Gallinarum var Pullorum | [1],9,12:-:- | eBG4 | ST92, ST747 |
| Enteritidis diphasic | 9,12:g,m:1,7 | ST746 | ST746 |
| Dublin | [1],9,12,[Vi]:g,p:- | eBG53, eBG93, eBG32 | ST10 plus 3 STs, ST180, ST74 |
| Rostock | [1],9,12:g,p,u:- | eBG53 | ST10 |
| Kiel | [1],2,12:g,p:- | eBG53 | ST10 |
| Naestved | [1],9,12:g,p,s:- | eBG53 | ST10 |
