# Supplementary material for: Multilocus Sequence Typing as a Replacement for Serotyping in Salmonella enterica
Source: PLoS Pathog. 2012 Jun 21;8(6):e1002776. doi: 10.1371/journal.ppat.1002776 (PMC3380943; doi:10.1371/journal.ppat.1002776)
Supplement: Table S5 — Antigenic formulas, eBGs, STs and dTar status of serovars associated with Paratyphi B. (DOC) [file ppat.1002776.s013.doc]

Supplementary Table 5. Antigenic formulas, eBGs, STs and dTar status of serovars associated with Paratyphi B

| Serovar | Antigenic formula | eBG | ST | dTar |
| --- | --- | --- | --- | --- |
| Paratyphi B | [1],4,[5],12,[27]:b:1,2 | eBG5 | ST86, ST265-267, ST772 | - |
| Paratyphi B monophasic | [1],4,[5],12:b:- | eBG32; eBG5 | ST42, ST86, ST264 | - |
| Paratyphi B var Java | [1],4,[5],12:b:1,2 | eBG5; eBG19; eBG59 | ST43 plus 6 STs; ST149, ST88 plus 2 STs; ST28 | + |
| Paratyphi B var Java monophasic | [1],4,[5],12:b:- | eBG19; eBG32; eBG155, ST135 | ST88, ST127; ST42, ST423, ST681, ST733; ST734, ST404, ST679, ST135 | + |
| *salamae* 4:12:b:- | 4,12:b:- | eBG214 | ST53, ST276 | + |
