# Supplementary material for: Multilocus Sequence Typing as a Replacement for Serotyping in Salmonella enterica
Source: PLoS Pathog. 2012 Jun 21;8(6):e1002776. doi: 10.1371/journal.ppat.1002776 (PMC3380943; doi:10.1371/journal.ppat.1002776)
Supplement: Text S1 — Deep phylogenetic structure and historical information regarding 6,7:c:1,5 isolates. (DOCX) [file ppat.1002776.s017.docx]

**Supplementary Note**

**Deep phylogenetic structure.** Although deep phylogenetic structure is difficult to determine in subspecies *enterica*, population genetic clustering has identified a cluster of related isolates called Clade B (now Lineage 3) on the basis of MLST sequences [1,2] and the analyses of numerous gene fragments [3,4]. We mapped Lineage 3 onto the MSTree with BAPS 5.3 on the basis of allelic differences among all 1097 STs (Fig. S1). BAPS analysis was performed using the clustering algorithm for unlinked loci with an upper-bound (K) for the number of clusters set in different runs for values between 2 and 7. The model probability increased only moderately between K values of 5 through 7, with a difference in frequency of assignment to Lineage 3 of only 2 %. Fig. S1 shows the results for K = 5. Similar results were obtained by the analysis of concatenated sequences from all seven genes with BAPS independent of K values up to 500 or STRUCTURE [5] with K = 5 (data not shown). The assignments to Lineage 3 by prior publications [1-4] were also consistently assigned to Lineage 3 by these results..

The MSTree in Fig. S1 show that almost all Lineage 3 STs cluster at the bottom of the MSTree, and that most other isolates belong to a second cluster, or to additional smaller clusters, which need to be resolved by more detailed analyses once additional genomic sequences are available. This higher order, deep phylogenetic structure is not as detailed as either serotyping or MLST. For example, lineage 3 currently includes 35 eBGs plus 126 singleton STs, as indicated in Table S2. It contains isolates from 137 of the 555 serovars tested here, including serovars Montevideo, Oranienburg, Pomona, Brandeburg, Schwarzengrund and Javiana. 33 of the 137 serovars within lineage 3 are also found outside of it, including serovars Montevideo and Oranienburg.

**Historical information regarding 6,7:c:1,5 isolates.** Isolates with the antigenic formula, 6,7:c:1,5 (serovars Choleraesuis, Paratyphi C, Typhisuis) were thought to be adapted to different animal hosts, and are sub-differentiated by biochemical tests [6] (Table 2). This sub-differentiation is difficult, even when carried out in well equipped reference facilities because biotyping is based on unstable phenotypic traits [7]. Currently there is considerable confusion over the exact host range and clinical phenotype of strains within the 6,7:c:1,5 group. Paratyphi C is described as causing “enteric fever” in humans, but a report exists that it was isolated from other animals [6]. Literature from the early 1900’s suggests that *S*. Paratyphi C causes a very mild form of illness in healthy adults except in the presence of malaria, where it can cause very severe septicaemia [8]. In contrast, Typhi and Paratyphi A are associated with the prolonged fever of classic enteric fever [9]. Typhisuis has only been described in pigs, where it causes the systemic infection called chronic swine para-typhoid, but only few reports exist and these bacteria are very rare [10].

Three biotyping variants have been described within Choleraesuis [6]: Choleraesuis itself (here designated as *s.s.* for *Sensu stricto*), Choleraesuis var. Kunzendorf and Choleraesuis var. Decatur (Table 3). All variants were thought to infect a wide range of hosts [11], and can cause a highly invasive disease in humans (especially the elderly and immuno-compromised) with only minor involvement of the gastro-intestinal tract [12,13]. Prior attempts to clarify the classification of the 6,7:c:1,5 group of *S. enterica* on the basis of MLEE [14,15] or IS200 patterns plus ribotyping [7] , has indicated that this group is more variable than other serovars and that biotyping does not represent a natural classification.

References

1. Falush D, Torpdahl M, Didelot X, Conrad DF, Wilson DJ et al. (2006) Mismatch induced speciation in *Salmonella*: model and data. Philos Trans R Soc Lond B Biol Sci 361: 2045-2053 .

2. Parsons SK, Bull CM, Gordon DM (2011) Substructure within *Salmonella enterica* subspecies *enterica* isolated from Australian wildlife. Appl Env Microbiol AEM .

3. Didelot X, Bowden R, Street T, Golubchik T, Spencer C et al. (2011) Recombination and population structure in *Salmonella enterica*. PLoS Pathog 7: e1002191 .

4. den Bakker HC, Moreno Switt AI, Govoni G, Cummings CA, Ranieri ML et al. (2011) Genome sequencing reveals diversification of virulence factor content and possible host adaptation in distinct subpopulations of *Salmonella enterica*. BMC Genomics 12: 425 .

5. Falush D, Stephens M, Pritchard JK (2003) Inference of population structure using multilocus genotype data: linked loci and correlated allele frequencies. Genetics 164: 1567-1587 .

6. Le Minor L, Beaud R, Laurent B, Monteil V (1985) *Salmonella* possessing the 6,7:c:1,5 antigenic factors. Ann Inst Pasteur Microbiol 136B: 225-234 .

7. Uzzau S, Hovi M, Stocker BA (1999) Application of ribotyping and IS*200* fingerprinting to distinguish the five *Salmonella* serotype O6,7:c:1,5 groups: Choleraesuis *sensu stricto*, Choleraesuis var. Kunzendorf, Choleraesuis var. Decatur, Paratyphi C, and Typhisuis. Epidemiol Infect 123: 37-46 .

8. Giglioli G (1933) Agglutinins for the Typhoid-Paratyphoid group in a random sample of the population of British Guiana. J Hyg 33: 379-386 .

9. Parry CM, Hien TT, Dougan G, White NJ, Farrar JJ (2002) Typhoid fever. N Engl J Med 347: 1770-1782 .

10. Uzzau S, Brown DJ, Wallis T, Rubino S, Leori G et al. (2000) Host adapted serotypes of *Salmonella enterica*. Epidemiol Infect 125: 229-255 .

11. Chiu CH, Su LH, Chu C (2004) *Salmonella enterica* serotype Choleraesuis: epidemiology, pathogenesis, clinical disease, and treatment. Clin Microbiol Rev 17: 311-322 .

12. Cohen JI, Bartlett JA, Corey GR (1987) Extra-intestinal manifestations of salmonella infections. Medicine (Baltimore) 66: 349-388 .

13. Chen PL, Wu CJ, Chang CM, Lee HC, Lee NY et al. (2007) Extraintestinal focal infections in adults with *Salmonella enterica* serotype Choleraesuis bacteremia. J Microbiol Immunol Infect 40: 240-247 .

14. Selander RK, Beltran P, Smith NH, Helmuth R, Rubin FA et al. (1990) Evolutionary genetic relationships of clones of *Salmonella* serovars that cause human typhoid and other enteric fevers. Infect Immun 58: 2262-2275 .

15. Boyd EF, Wang FS, Beltran P, Plock SA, Nelson K et al. (1993) *Salmonella* reference collection B (SARB): strains of 37 serovars of subspecies I. J Gen Microbiol 139: 1125-1132 .
